# Distinct prediction errors in mesostriatal circuits of the human brain mediate learning about the values of both states and actions: evidence from high-resolution fMRI

**DOI:** 10.1371/journal.pcbi.1005810

**Published:** 2017-10-19

**Authors:** Jaron T. Colas, Wolfgang M. Pauli, Tobias Larsen, J. Michael Tyszka, John P. O’Doherty

**Affiliations:** 1 Computation and Neural Systems Program, California Institute of Technology, Pasadena, CA, United States of America; 2 Division of the Humanities and Social Sciences, California Institute of Technology, Pasadena, CA, United States of America; 3 Center for Mind/Brain Sciences, University of Trento, Trento, Italy; Harvard University, UNITED STATES

## Abstract

Prediction-error signals consistent with formal models of “reinforcement learning” (RL) have repeatedly been found within dopaminergic nuclei of the midbrain and dopaminoceptive areas of the striatum. However, the precise form of the RL algorithms implemented in the human brain is not yet well determined. Here, we created a novel paradigm optimized to dissociate the subtypes of reward-prediction errors that function as the key computational signatures of two distinct classes of RL models—namely, “actor/critic” models and action-value-learning models (e.g., the Q-learning model). The state-value-prediction error (SVPE), which is independent of actions, is a hallmark of the actor/critic architecture, whereas the action-value-prediction error (AVPE) is the distinguishing feature of action-value-learning algorithms. To test for the presence of these prediction-error signals in the brain, we scanned human participants with a high-resolution functional magnetic-resonance imaging (fMRI) protocol optimized to enable measurement of neural activity in the dopaminergic midbrain as well as the striatal areas to which it projects. In keeping with the actor/critic model, the SVPE signal was detected in the substantia nigra. The SVPE was also clearly present in both the ventral striatum and the dorsal striatum. However, alongside these purely state-value-based computations we also found evidence for AVPE signals throughout the striatum. These high-resolution fMRI findings suggest that model-free aspects of reward learning in humans can be explained algorithmically with RL in terms of an actor/critic mechanism operating in parallel with a system for more direct action-value learning.

## Author summary

An accumulating body of evidence suggests that signals of a reward-prediction error encoded by dopaminergic neurons in the midbrain comprise a fundamental mechanism underpinning reward learning, including learning of instrumental actions. Nevertheless, a major open question concerns the specific computational details of the “reinforcement-learning” algorithms through which these prediction-error signals are generated. Here, we designed a novel task specifically to address this issue. A fundamental distinction is drawn between predictions based on the values of states and predictions based on the values of actions. We found evidence in the human brain that different prediction-error signals involved in learning about the values of either states or actions are represented in the substantia nigra and the striatum. These findings are consistent with an “actor/critic” (i.e., state-value-learning) architecture updating in parallel with a more direct action-value-learning system, providing important constraints on the actual form of the reinforcement-learning computations that are implemented in the mesostriatal dopamine system in humans.

## Introduction

Efforts to achieve a computational-level understanding of how the brain learns to produce adaptive behavior from rewarding and punishing feedback have gained inspiration from a class of abstract models falling under the umbrella of “reinforcement learning” (RL) with roots in machine learning and artificial intelligence [1–3] as well as psychology [4]. Intense focus on the applicability of these models to actual nervous systems arose following the seminal finding that the phasic activity of dopaminergic neurons within the midbrain—in particular, the substantia nigra (SN) and the ventral tegmental area (VTA)—resembles a reward-prediction-error (RPE) signal from the temporal-difference (TD) algorithm [5] characteristic of a number of such RL models [6–11].

Yet, a major open question in the literature concerns the precise form of the RL algorithm or algorithms that the brain—and, in particular, the mesostriatal dopamine system—deploys. The “actor/critic” model [12–14] represents one class of RL algorithms that has been put forth to account for the functional neurocircuitry of reward learning in the basal ganglia [6,15–20]. Evoking the classical two-process theory of instrumental [21] and Pavlovian [22]—essentially, response-dependent and response-independent—conditioning [23,24], the actor/critic theory postulates that two distinct modules play a role: the “critic” learns about the values of states independently of the actions taken in those states, whereas the “actor” is involved in encoding the action policy—that is, the likelihood of taking a particular action in a given state. The TD error is computed using the state-value predictions generated by the critic, and this same error signal is then used to update the policy in the actor module proposed. Evidence supporting an actor/critic architecture in the brain has emerged from observations illustrating a broad dorsal-ventral distinction in the functions of the striatum: the ventral striatum (i.e., the ventral putamen and the nucleus accumbens) is dedicated to learning and encoding reward predictions without regard for actions, whereas the dorsal striatum (i.e., the dorsal putamen and the caudate nucleus) is more involved for situations in which actions are learned and selected in order to obtain rewards [25–28]. In keeping with the actor/critic framework, the ventral striatum has been found to encode RPE signals during passive reward learning (i.e., Pavlovian conditioning) as well as active reward learning (i.e., instrumental conditioning), whereas the dorsal striatum has more typically been reported to be selectively engaged for instrumental-learning paradigms in which actions must be selected to obtain rewards [19,29–31].

However, the actor/critic model offers but one of several RL-based accounts for learning representations of hedonic value and instrumental behavior. Another class of models known here as action-value-learning models [32,33] even dispenses with learning about the values of states altogether and instead learns directly about the values of specific actions available within each given state. Thus, the corresponding TD prediction error is computed in accordance with differences in successive predictions about the values of actions as opposed to states. In simulations where the action space is tractably small and well delineated, an action-value-learning model such as the Q-learning model [32] is reported to converge more quickly than the actor/critic model, which indicates that the former class of models is generally more efficient for learning actions [3].

Given that actor/critic and action-value-learning variants of RL models make qualitatively divergent predictions about the nature of the TD-learning error signal [34], it is perhaps surprising that, to date, only a handful of studies have attempted to directly ascertain which algorithm best accounts for neural activity in dopaminergic regions during instrumental-learning tasks [8,9,35,36]. Moreover, studies have yielded differing conclusions with discrepancies further complicated by differences in species, recording sites, and tasks across studies: evidence from Morris and colleagues [8] suggested that an action-value-learning algorithm is implemented in the substantia nigra pars compacta (SNc) in macaque monkeys, whereas Roesch and colleagues [9] presented evidence in the VTA in rats consistent with either an action-value-learning algorithm or an actor/critic scheme.

**Fig 1:**
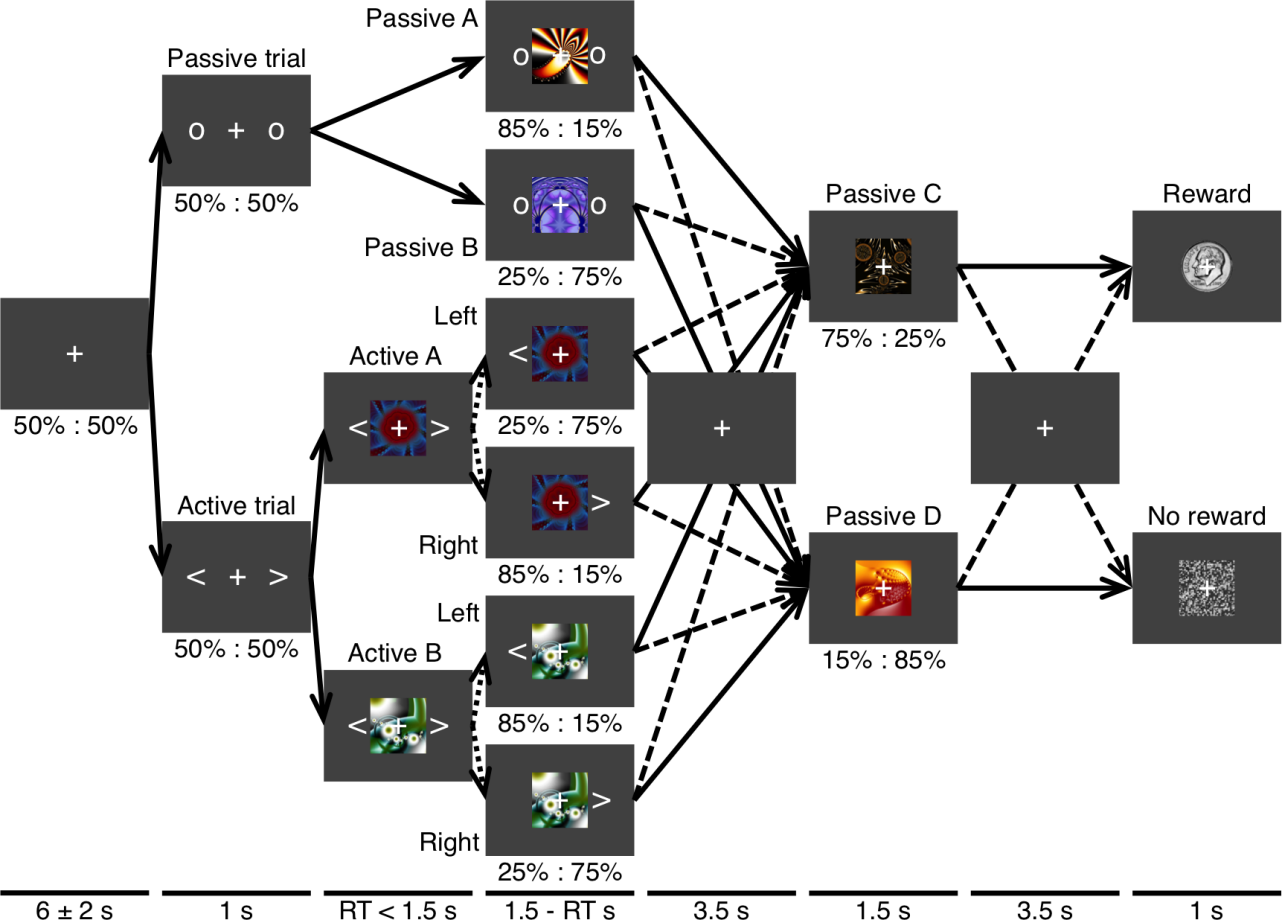
Markov decision process. This schematic of the task illustrates the transition probabilities for a Markov decision process featuring interleaved and interrelated passive and active states. Passive and active types of trials occurred with equal probability. On a passive trial the initial presentation of two circles was followed by one of two fractal cues that each represented a first-stage passive state. The transition probabilities for the first-stage state determined which of two second-stage passive states (i.e., fractals) were to be presented next. The transition probabilities for the two second-stage states determined whether the final outcome was a monetary reward or nothing. On an active trial, two arrows were followed by one of two fractals that each represented a first-stage active state. The transition probabilities for an action given the state determined which of the same pair of second-stage passive states was to be presented next. Solid lines represent transitions having an equal or relatively greater probability of occurring. Dashed lines represent transitions having a relatively lower probability of occurring. Dotted lines represent transitions that are determined by an action. The fixation cross appeared as depicted on every trial regardless of whether a given arrow actually passes through the representation of an interstimulus interval here.

The primary goal of the present study was to compare and contrast the actor/critic model and action-value-learning models, which are both theoretically sound implementations of RL, with an aim to best capture activity in the dopaminergic midbrain and dopaminoceptive target areas of the striatum in humans by identifying the specific features of the prediction-error codes in these structures. To achieve this, we scanned the brains of human subjects with fMRI while they attempted to learn about a multi-step Markov decision process (MDP) (**Fig 1**). This unique task was specifically designed to enable us to distinguish two possible manifestations of the RPE signal—namely, a state-value-prediction error (SVPE), which would be produced by an actor/critic-like mechanism in which prediction errors are computed by comparing successive differences in state values (i.e., the value of being in a particular state regardless of actions), and an action-value-prediction error (AVPE), which would be computed by comparing successive predictions for the values of specific actions as per an action-value-learning algorithm such as Q learning.

An inherent challenge in dissociating state values and action values is that they tend to be highly correlated with each other in most instrumental-learning settings. Thus, prior to programming the fMRl experiment presented herein, we first ran extensive simulations in order to refine the parameters of the MDP and obtain an optimal task design that allowed for maximal separation of estimated state values and action values as simulated by RL model variants. A key feature of our task and the MDP that enabled us to achieve the necessary decoupling is that, while some states required selection of an action in order to transition to a new state, other states did not have any actions available and instead would result in the observer passively transitioning from one state to another. Importantly, not only were interleaved passive states differentially associated with receiving subsequent rewards, but it was also the case that intermediate passive states could be reached by either transitioning passively or taking particular actions. Participants thus needed to learn about the values of both active and passive states in order to most effectively solve the task. This configuration is ideal in that both state-value learning and action-value learning can take place and generate the respective signature signals of these variants of reward learning.

Of note is that, although previous attempts to probe the SVPE in isolation have relied on Pavlovian-conditioning paradigms for which there is ostensibly no instrumental action-based component (e.g., [19,37,38]), the signals observed for strictly Pavlovian learning paradigms cannot unambiguously address the nature of the RL signals invoked during instrumental-learning paradigms. This limitation follows from the fact that it is entirely plausible that there exists a separate Pavlovian value-learning system acting independently from the system dedicated to learning about the values of instrumental actions. Another relevant factor is that it can be difficult to completely rule out the roles of incidental actions simultaneously present during Pavlovian learning that could actually be instrumentally controlled, such as voluntary eye movements, oral actions (for gustatory rewards), or instrumental approach behaviors. The new approach explored here overcomes those issues inasmuch as state-value learning and action learning were both embedded in the same integrated instrumental-learning paradigm, such that the respective signals can be juxtaposed directly as they are potentially computed in parallel.

To enable us to effectively resolve blood-oxygen-level-dependent (BOLD) activity within the midbrain’s dopaminergic nuclei in the midbrain—which poses additional technical challenges [39–41]—we employed a high-resolution functional magnetic-resonance imaging (fMRI) protocol with 1.5-mm isotropic voxels that was optimized for the midbrain and the striatum (see [38] for a similar approach). As part of this protocol, we concurrently measured cardiac and respiratory activity and then used these physiological signals to account for contaminating effects of physiological noise in the fMRI data, which is particularly detrimental to image quality in the tegmentum [42–44]. Furthermore, we deployed a specialized preprocessing pipeline that included denoising of the images and was also developed to optimize between-subject alignment of mesencephalic structures. The field of view for this imaging protocol could accommodate both ventral and dorsal portions of the striatum and even parts of ventromedial prefrontal cortex (vmPFC) for its role in computing value signals [30,45,46]. Hence, high-resolution functional images were obtained from both the dopaminergic midbrain and its striatal target regions.

Neuroanatomical evidence points to different subregions of the dopaminergic midbrain as having distinct projections to target areas of the striatum: dopaminergic neurons in the dorsal tier comprising the VTA and the dorsal SNc project to more ventral areas of the striatum, whereas dopaminergic neurons in the ventral tier including most of the SNc project to more dorsal areas of the striatum [27,47–49]. In light of this anatomical dissociation, we hypothesized that the two distinct subtypes of the RPE signal would be encoded within different subregions of the dopaminergic midbrain. Yet, even at this maximal spatial resolution, precisely delineating the dopaminergic tiers or even the SNc as a whole within the SN is beyond the capabilities of these methods [50]. Specifically, we hypothesized that the VTA and some parts of the SN would be more involved in encoding the critic module’s SVPE, while other parts of the SN would be more involved in encoding the AVPE computed by an action-value-learning algorithm. We additionally expected to find evidence of SVPE signals in the ventral-striatal areas targeted by the dorsal tier of the dopaminergic midbrain as well as evidence of AVPE signals in the dorsal-striatal areas instead principally linked with the ventral tier.

As a secondary aim, we also set out to replicate findings from Schönberg and colleagues [51] that RPE-related activity in the dorsal striatum alone would distinguish subjects according to the degree of learning as assessed behaviorally. Elaborating further on the original findings of Schönberg and colleagues [51] by virtue of the unique capabilities of the current paradigm, we also hypothesized that such a relationship between brain and behavior would be observed with respect to an AVPE signal in particular.

## Results

### Behavioral performance

Following a similar approach taken by Schönberg and colleagues [51], participants were first divided into two groups according to their behavioral performance on the task (**Table 1**). Of 39 total subjects, 20 were individually classified as “Good-learner” subjects for whom choice accuracy was significantly greater than the chance level of 50% (*p* < 0.05 according to a binomial test). The remaining 19 participants for whom the null hypothesis of chance accuracy could not be rejected with significance at the individual level were further subdivided into 15 “Poor-learner” subjects, who nonetheless could be accounted for with an RL model, and only 4 “Nonperformer” subjects, who were excluded from further analysis because subsequent computational modeling determined that the behavior of these individuals was completely insensitive to outcomes. Whereas the Good-learner and Poor-learner groups were defined on the basis of differences in accuracy, there were no significant differences between the groups when considering possible confounds in reaction time (RT), errors such as missed responses or inappropriate responses that resulted in missed trials, or the demographic variables of age and gender (*p* > 0.05) (**Table 1**). Accuracy was significantly greater than the chance level across subjects not only within the Good-learner group (*M* = 20.9%, *t*_*19*_ = 13.22, *p* < 10^-10^) but also within the Poor-learner group (*M* = 3.1%, *t*_*14*_ = 2.23, *p* = 0.021) despite not having sufficient statistical power to verify the effects for Poor learners at the individual level. These results and model fitting together demonstrate that, unlike the Nonperformers, the Poor learners made an effort to attend to and perform the task and, in doing so, did in fact learn—albeit to a lesser extent than the Good learners.

**Table 1:** Subject groups. Subjects were first objectively divided into two groups a priori according to their performance on the task as represented by the accuracy score listed here. Of 39 total subjects, 20 were classified as “Good-learner” subjects for whom choice accuracy was significantly greater than the chance score of 50% at the level of an individual subject (*p* < 0.05). Of the remaining 19 “Poor-learner” subjects, 4 were subsequently reclassified as “Nonperformer” subjects in cases of complete insensitivity to outcomes, which was verified with computational modeling. There were no significant differences between the two main groups when considering possible confounds in reaction time (RT), the total number of missed trials following errors, or age and gender (*p* > 0.05). Standard deviations are listed in parentheses by the corresponding means within groups.

	Good learner	Poor learner	Nonperformer	Performer	Aggregate
*n*	20	15	4	35	39
Accuracy (%)	70.9 (7.1)	53.1 (5.4)	43.5 (6.1)	63.3 (10.9)	61.2 (12.1)
RT (ms)	755 (107)	779 (137)	712 (170)	765 (120)	760 (124)
Missed trials	6.0 (5.2)	5.5 (5.3)	12.8 (13.1)	5.8 (5.2)	6.5 (6.5)
Age (y)	23.5 (3.8)	25.8 (5.2)	27.3 (8.3)	24.5 (4.6)	24.7 (5.0)
M:F (%)	50	40	100	45.7	51.3

### Behavioral model fitting

We considered as a possibility not only “model-free” (i.e., habitual) learning [21,22] but also “model-based” (i.e., goal-directed) learning [52]. Thus, four computational modules—to wit, the critic component of the actor/critic (i.e., a state-value learner), the actor component of the actor/critic guided by the critic, an action-value learner, and a model-based learner—were tested along with combinations of these. We first implemented the standard actor/critic model [12–14], which updates both the critic’s cached state values and the actor’s policy via a common SVPE, and the Q-learning model [32], a canonical action-value-learning model that forgoes state values to instead directly encode action values that are updated via an AVPE. As these model-free alternatives are not mutually exclusive but rather could each exist as part of parallel systems within the brain, we took the novel approach of hybridizing them. In the presence of passive states, the “critic/Q-learner” (CQ) model, which is again a TD model, integrates the state-value predictions of the critic into the action-value updates that exclusively determine the action policy. The “actor/critic/Q-learner” (ACQ) model goes a step further to fully integrate the SVPE and the AVPE into the action weights actually driving behavior. We also tested a model-based (MB) model with a dynamic-programming algorithm [3,53,54] by which the agent learns the transitions from state-action pairs and utilizes knowledge of the transition functions and reward availability to compute action-value estimates on the fly. This MB model was likewise incorporated into hybrid models that paired model-based learning with each of the four aforementioned variants of model-free learning. The hybrid models integrated the outputs of each individual algorithm to compute net action weights according to static input-weighting parameters, which were fitted along with other free parameters at the level of individual subjects. Additional details about the models and model-fitting procedures are provided in the Methods section.

**Fig 2:**
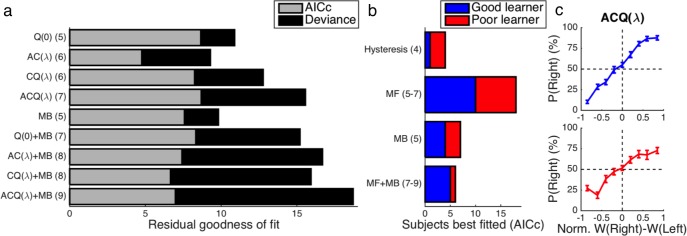
Model fitting and behavior. **(a)** Average goodness of fit relative to the outcome-insensitive hysteresis model across performing subjects is shown for each model tested with (light bars) and without (light and dark bars combined) a penalty for model complexity according to the AICc. A positive residual corresponds to a superior fit. After correcting for model complexity, the 7-parameter ACQ model provided the best overall fit for the data. Degrees of freedom are listed in parentheses. **(b)** At the level of individual subjects, the AICc generally favored a model-free (MF) algorithm as opposed to a model-based (MB) algorithm or some combination of the two within both the Good-learner group (blue bars) and the Poor-learner group (red bars). **(c)** The relationship between the normalized difference in the net action weights *W*_*t*_*(s*_*t*_,*a)* predicted by the ACQ model and observed choices is plotted separately for the Good-learner (blue line) and Poor-learner (red line) groups. Error bars indicate standard errors of the means.

Each subject was modeled separately in a factorial model comparison with 22 alternatives that simultaneously assessed model-free learning in its various forms, model-based learning, and “TD(λ)” eligibility traces as a potential augmentation of model-free learning—all while rigorously controlling for internal choice biases and hysteresis. While noting the caveat that model-free TD(λ) learning requires one more degree of freedom than model-based learning with its assumptions that are actually less parsimonious but unquantifiable as such, formal penalties for model complexity were imposed according to the Akaike information criterion with correction for finite sample size (AICc) [55,56]. Taking into account all of the performing subjects (i.e., Good learners and Poor learners) collectively to maximize not only statistical power but also generalizability, the 7-parameter “ACQ(λ)” model (henceforth abbreviated as “ACQ”) was found to provide the best account of behavioral choice data among the candidate models (**Fig 2A**). For this reason, the ACQ model was utilized in the subsequent fMRI analyses reported here. When considering fits at the level of individual subjects, model-free learning with an eligibility trace available was also found to be generally preferred to the MB model or a model-free/model-based hybrid after formally penalizing model complexity (**Fig 2B**). Details of the ACQ model’s fitted parameters are provided in **Table 2**.

**Table 2:** Model parameters. The means and standard deviations of the ACQ model’s fitted parameters—including from the hysteresis model the (arbitrarily rightward) constant choice bias *β*_*R*_ and initial magnitude *β*_*0*_ coupled with inverse decay rate *λ*_*β*_ for exponential decay of the perseveration bias—are listed separately for each group, revealing a tendency for Good learners to have lower temperature than Poor learners (*M* = 0.987, *t*_*33*_ = 2.88, *p* = 0.004). The logarithm of the ratio between the eligibility-adjusted learning rate and the temperature provides a more precise metric for the sensitivity dictated by the model’s fitted parameters than the temperature alone—especially given the correlation between the eligibility-adjusted learning rate and the temperature [57] exhibited within the Poor-learner group (*r* = 0.547, *t*_*13*_ = 2.36, *p* = 0.035) and the lack of such a correlation among Good learners (*r* = 0.121, *t*_*18*_ = 0.52, *p* = 0.611). Model sensitivity, which was significantly positive across the Good-learner group (*M* = 0.440, *t*_*19*_ = 5.59, *p* < 10^-4^) but not the Poor-learner group (*M* = 0.020, *t*_*14*_ = 0.18, *p* = 0.428), was not only greater for Good learners than for Poor learners (*M* = 0.420, *t*_*33*_ = 3.23, *p* = 10^-3^) but also significantly correlated with the objective metric for choice accuracy (*r* = 0.409, *t*_*33*_ = 2.57, *p* = 0.007). The residual deviance *D* (with degrees of freedom in the subscript) corresponds to the ACQ model’s improvement in fit relative to either a null intercept model or the hysteresis model.

	Good learner	Poor learner
*n*	20	15
Accuracy (%)	70.9 (7.1)	53.1 (5.4)
Sensitivity *log(α(1+λ)/τ)*	0.440 (0.352)	0.020 (0.417)
Learning rate *α*	0.588 (0.237)	0.551 (0.308)
Eligibility *λ*	0.682 (0.323)	0.687 (0.431)
Action-value weight *w*_*Q*_	0.661 (0.315)	0.626 (0.418)
Softmax temperature *τ*	0.404 (0.262)	1.390 (1.512)
Perseveration bias: magnitude *β*_*0*_	0.093 (0.366)	-0.088 (0.521)
Perseveration bias: rate *λ*_*β*_	0.621 (0.375)	0.751 (0.281)
Rightward bias *β*_*R*_	0.230 (0.425)	0.128 (0.673)
Null: residual deviance *D*_*6*_	45.60 (20.31)	21.59 (20.15)
Hysteresis: residual deviance *D*_*3*_	20.18 (13.32)	9.41 (9.13)

An important caveat of the model comparison at the group level is that, after adjusting for model complexity, the ACQ model yielded only a marginally improved fit to behavior as compared to the simple Q-learning model (i.e., the “Q(0)” model). This suggests that the predictions of the hybrid ACQ model and the pure Q-learning model cannot be clearly separated on the basis of the behavioral data alone in the present study. Nevertheless, for the purpose of examining neural computations related to either state values or action values in the fMRI data, the ACQ model remains appropriate to use inasmuch as it enables us to simultaneously test for both forms of value signals along with their respective prediction-error signals. For the sake of completeness, we also used the Q(0) model as part of another computational-model-based analysis of the fMRI data, which is discussed briefly below.

The probability of an action increased in an orderly fashion with the difference between its net action weight as predicted by the ACQ model and the net weight of the alternative for both the Good-learner group and the Poor-learner group (**Fig 2C**), providing evidence for the quality of the model’s fits to the behavioral data. In a similar vein, we noted that RTs became faster as the absolute difference between net action weights increased for both the Good-learner group (*β* = 86 ms, *t*_*19*_ = 3.38, *p* = 0.002) and the Poor-learner group (*β* = 114 ms, *t*_*14*_ = 3.67, *p* = 0.001). Using logistic regression, we also found evidence for a bias in favor of repeating the previous action given the current state in both Good learners (*β* = 0.368, *t*_*19*_ = 2.66, *p* = 0.008) and Poor learners (*β* = 0.194, *t*_*14*_ = 2.08, *p* = 0.028), confirming that participants showed perseveration tendencies for previously performed actions [58] as in the computational model.

### Computational-model-based analysis of neuroimaging data

Applying the ACQ model to the fMRI data [59], we generated regressors corresponding to the prediction-error signals and value signals that were simulated explicitly (see Methods for details) (**S2 Fig**). In particular, we tested for neural activity correlating with the SVPE *δ*^*V*^_*t*_, which is produced by the critic component of the combined model, and the AVPE *δ*^*Q*^_*t*_, which is produced by the Q-learning component. The representations of the state value *V*_*t*_*(s*_*t*_*)* and the action value *Q*_*t*_*(s*_*t*_,*a*_*t*_*)* themselves were also examined. To assess these neurophysiological signals in relation to differences in behavioral performance, we analyzed the Good-learner and Poor-learner groups both separately and collectively and also directly tested for differences in effects between the two groups in an independent voxel-wise manner.

### All performing participants

We hypothesized that, during learning of the MDP, we would find evidence for separate SVPE and AVPE signals. Initially, effects of the SVPE and the AVPE were examined across all performing subjects as a whole, including both the Good-learner group and the Poor-learner group.

### State-value-prediction-error signals

**Fig 3:**
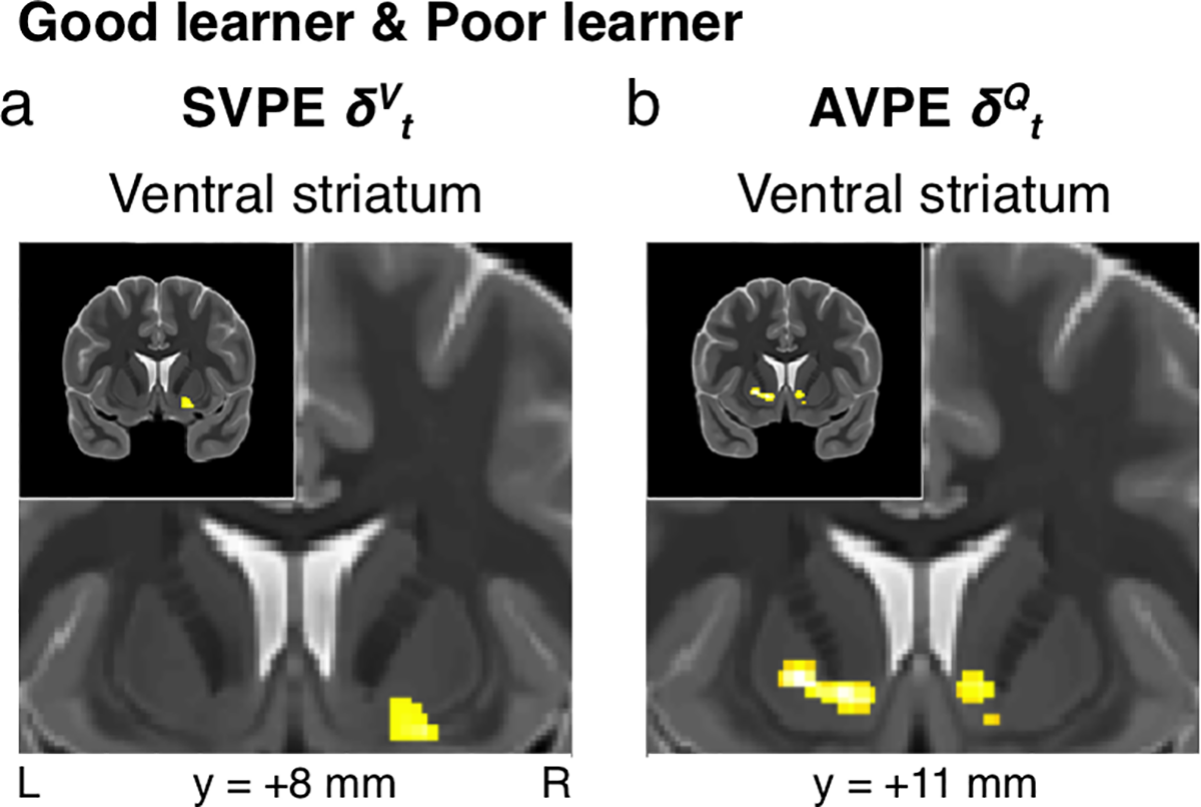
All performing participants: Two types of reward-prediction-error signals. **(a)** State-value-prediction error (SVPE) *δ*^*V*^_*t*_ signals were observed in the ventral striatum across all performing subjects (*p* < 0.005 unc., SVC *p*_*FWE*_ < 0.05). **(b)** Complementary action-value-prediction error (AVPE) *δ*^*Q*^_*t*_ signals were likewise identified in the ventral striatum (*p* < 0.005 unc., SVC *p*_*FWE*_ < 0.05). As in subsequent figures, the upper-left corner of each panel depicts the entire coronal section that the remainder of the respective panel zooms in on.

As expected in the striatal regions that the dopaminergic midbrain projects to, there was an SVPE signal in the right ventral striatum (*xyz* = [19, 12.5, -13], *t*_*34*_ = 4.09, *p* = 10^-4^, *k* = 69, SVC *p*_*FWE*_ < 0.05) (**Fig 3A**), including the ventral putamen and the nucleus accumbens. Although we did also find some effects of the SVPE in the left SN, the cluster did not fully reach the corrected threshold for significance (SVC *p*_*FWE*_ = 0.100).

### Action-value-prediction-error signals

As part of the same model, effects of the AVPE were also observed in the ventral striatum in both the left (*xyz* = [-12.5, 11, -5.5], *t*_*34*_ = 4.44, *p* < 10^-4^, *k* = 115, SVC *p*_*FWE*_ < 0.05) and the right (*xyz* = [8.5, 12.5, -4], *t*_*34*_ = 3.87, *p* < 10^-3^, *k* = 108, SVC *p*_*FWE*_ < 0.05) hemispheres (**Fig 3B**).

### Value signals

**Fig 4:**
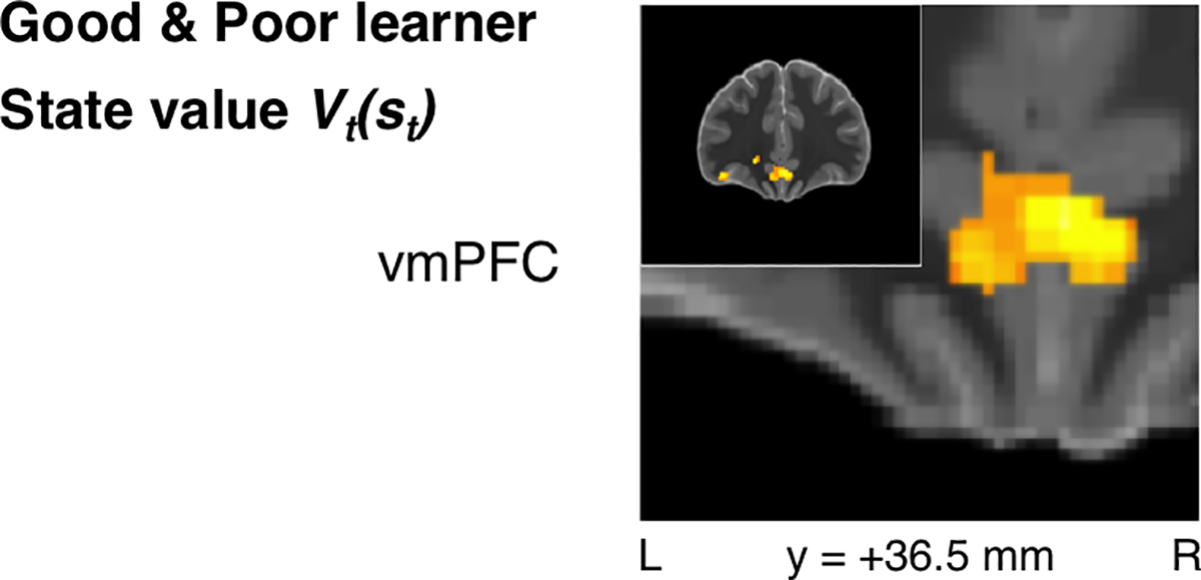
All performing participants: State-value signals. State value *V*_*t*_*(s*_*t*_*)* signals were observed in ventromedial prefrontal cortex (vmPFC) across all performing subjects as hypothesized (*p* < 0.005 unc., SVC *p*_*FWE*_ < 0.05).

vmPFC was also partially acquired within the current field of view despite it not extending all the way to the frontal pole. Accordingly, we were also able to test for the presence of value signals in this region; such signals have been reported consistently in prior literature and even demonstrated with meta-analyses [30,45,46]. In keeping with this prior literature, an aggregate analysis across all performing subjects yielded effects for state-value signals in vmPFC (*xyz* = [2.5, 35, -13], *t*_*34*_ = 4.86, *p* = 10^-5^, *k* = 399, SVC *p*_*FWE*_ < 0.05) (**Fig 4**). No significant effect of the AVPE was found across this pooled sample.

### Good-learner group

In order to examine effects specifically in those participants who learned the task successfully, we next focused on the Good-learner group alone.

### State-value-prediction-error signals

**Fig 5:**
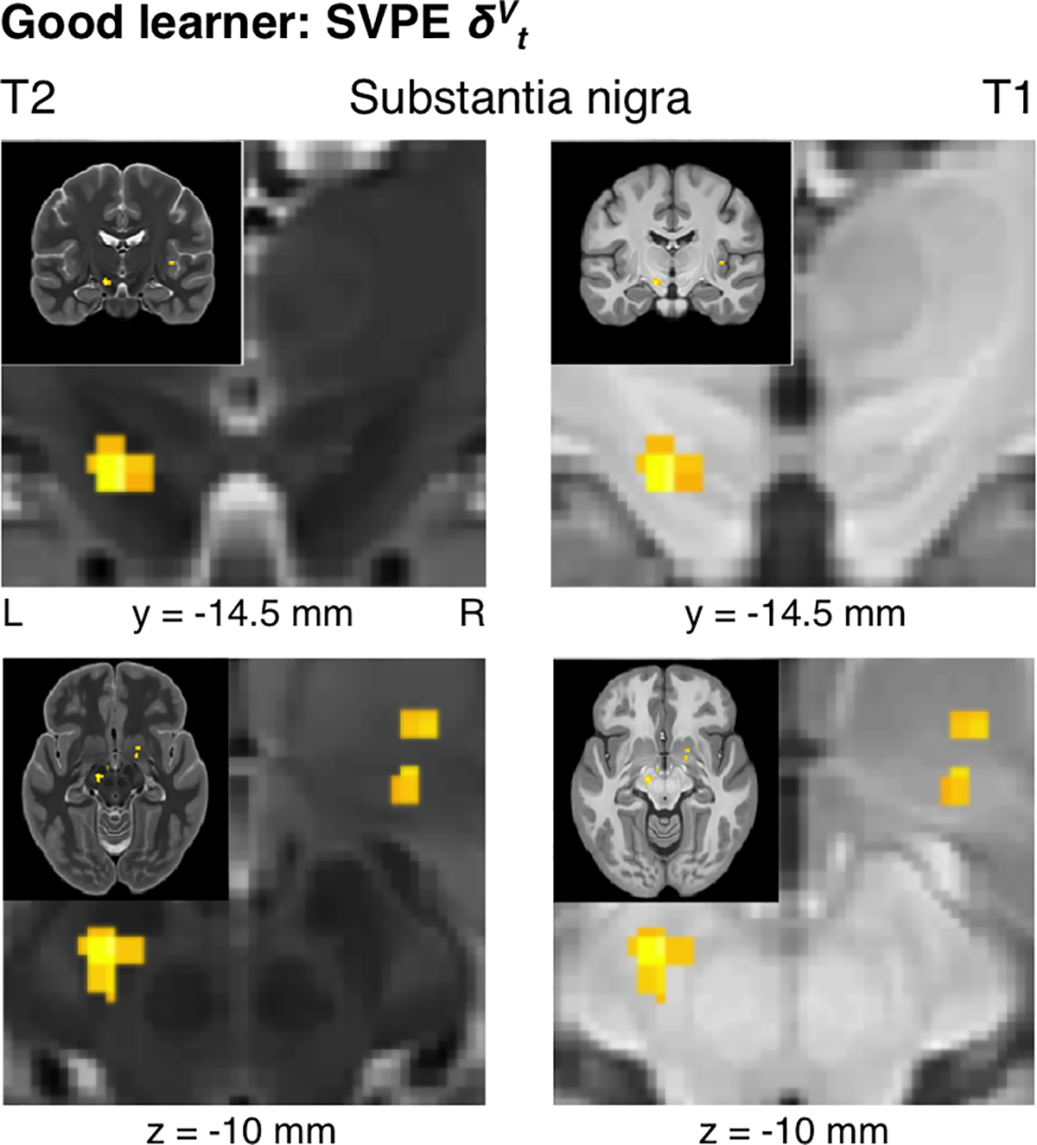
Good-learner group: State-value-prediction-error signals in the dopaminergic midbrain. Focusing on the dopaminergic midbrain, SVPE signals were found within the substantia nigra for the Good-learner group (*p* < 0.005 unc., SVC *p*_*FWE*_ < 0.05). To better visualize the anatomy of the dopaminergic midbrain, the same statistical map is plotted over T2-weighted and T1-weighted structural images in the left and right panels, respectively. Also visible is the ventral tegmental area (high intensity for T2, low intensity for T1), corresponding to a region between the dorsal edge of the substantia nigra (low intensity for T2, heterogeneous intensity for T1) and the ventral edge of the red nucleus (low intensity for T2, high intensity for T1). Coronal sections are displayed in the upper panels, and axial sections are displayed in the lower panels.

Our initial hypothesis concerning RPE signals in the dopaminergic midbrain was partly confirmed to the extent that significant SVPE signals were identified in the left SN for the Good learners (*xyz* = [-11, -14.5, -11.5], *t*_*19*_ = 4.32, *p* < 10^-3^, *k* = 26, SVC *p*_*FWE*_ < 0.05) (**Fig 5**). Importantly, these results were obtained with a model in which the AVPE was also entered as a parametric regressor so as to compete equally for variance alongside the SVPE. As a consequence of this feature, the present results show that SVPE-related activity in the substantia nigra can be accounted for by the SVPE signal after controlling for any effects of the AVPE in accordance with the extra-sum-of-squares principle. We also tested whether voxels in the dopaminergic midbrain responded to the SVPE to a significantly greater extent than to the AVPE by performing a direct contrast between the SVPE and AVPE regressors, but this contrast revealed no significant effects (*p* > 0.005). Thus, we cannot conclude that the SVPE provides a significantly better account of activity in this brain region. However, we can conclude that the SVPE-related activity found in this region is not accounted for by the AVPE up to the limits of the robustness of the statistical test.

**Fig 6:**
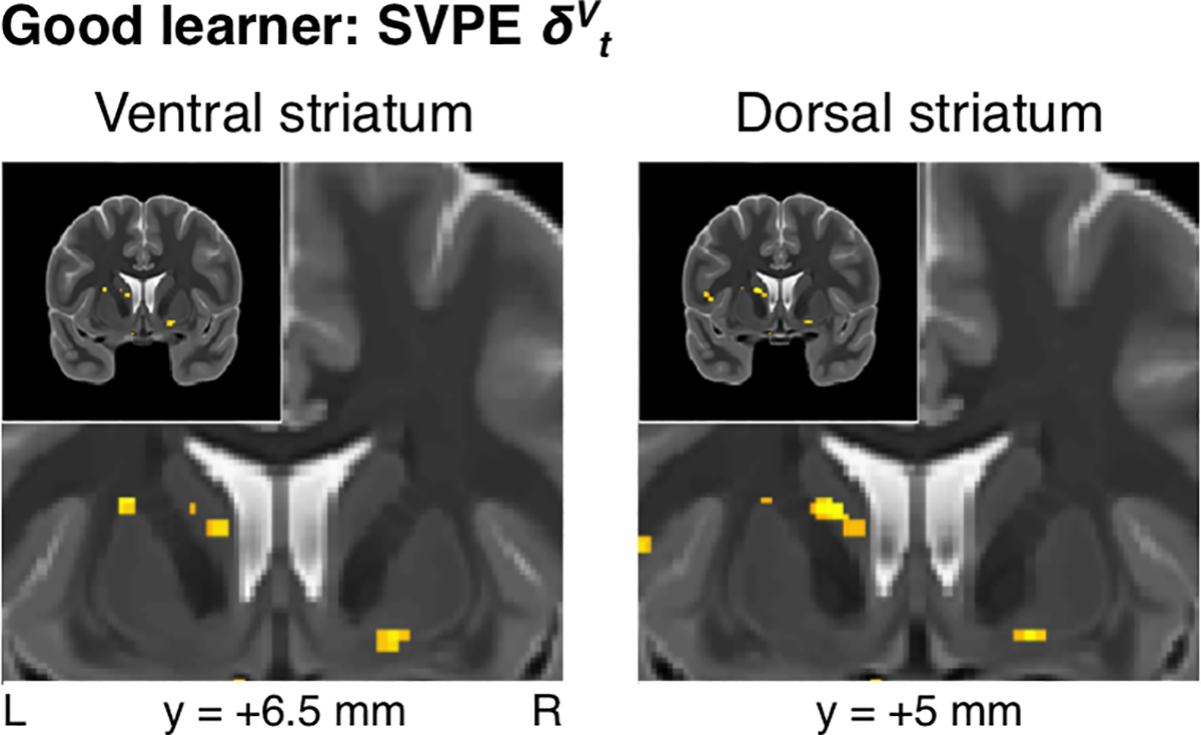
Good-learner group: State-value-prediction-error signals in the striatum. In addition to the substantia nigra, SVPE signals were also located in both the ventral striatum and the dorsal striatum for the Good-learner group (*p* < 0.005 unc., SVC *p*_*FWE*_ < 0.05).

In addition to revealing significant effects of the SVPE within the dopaminergic midbrain, we also tested for SVPE signals in the striatum. Consistent with the results from the pooled analysis across all performing participants, effects of the SVPE were found in the right ventral striatum (*xyz* = [17.5, 2, -8.5], *t*_*19*_ = 3.67, *p* < 10^-3^, *k* = 38, SVC *p*_*FWE*_ < 0.05) (**Fig 6**) for the Good learners alone. We also found evidence for SVPE signals in the left caudate nucleus within the dorsal striatum (*xyz* = [-17, 2, 15.5], *t*_*19*_ = 4.65, *p* < 10^-4^, *k* = 66, SVC *p*_*FWE*_ < 0.05) (**Fig 6**). Altogether, this mesostriatal network encoding the SVPE was significant at the set level across all regions of interest (ROIs) in the dopaminergic midbrain and the striatum (SVC *p*_*FWE*_ < 0.05).

### Action-value-prediction-error signals

AVPE signals were likewise identified in the striatum for the Good-learner group. Also found was an effect of the AVPE in the right ventral striatum (*xyz* = [8.5, 11, -2.5], *t*_*19*_ = 4.02, *p* < 10^-3^, *k* = 71, SVC *p*_*FWE*_ = 0.064) that borders but does not quite reach our significance threshold. This cluster also extended into the dorsal striatum, where its global peak was located (*xyz* = [11.5, 20, -2.5], *t*_*19*_ = 4.13, *p* < 10^-3^), and an anterior region of the caudate nucleus in close proximity to that originally reported for an instrumental RPE signal by O’Doherty and colleagues [19]. Additional clusters for the AVPE were observed throughout the dorsal striatum at an uncorrected threshold (**S3 Fig**).

### Value signals

**Fig 7:**
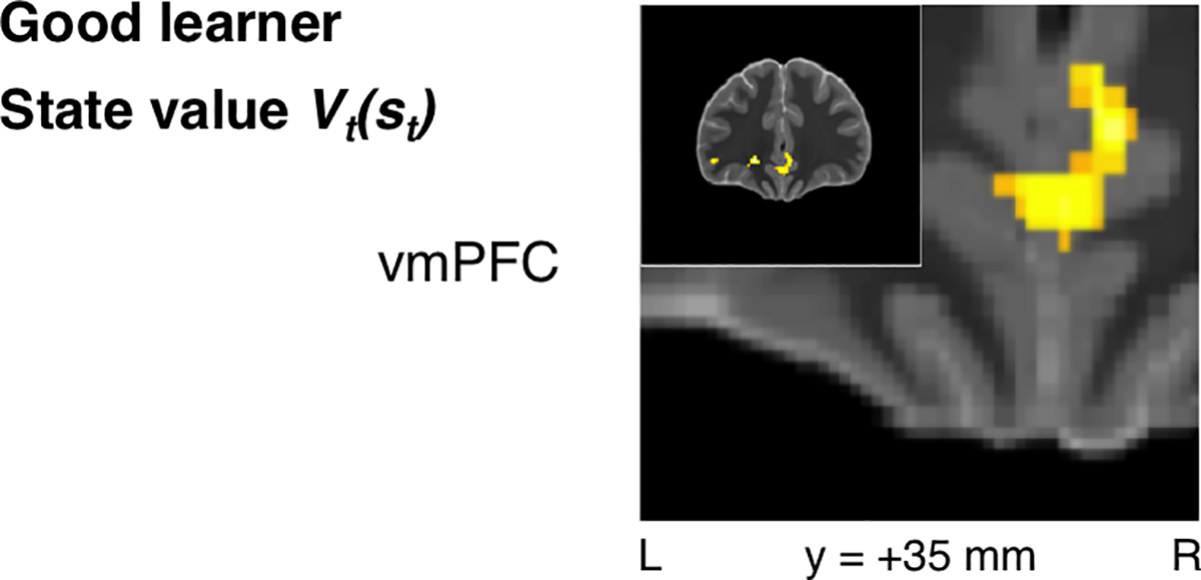
Good-learner group: State-value signals. State-value signals were similarly identified in vmPFC when focusing on the Good-learner group alone (*p* < 0.005 unc., SVC *p*_*FWE*_ < 0.05).

State-value signals were significant in bilateral vmPFC (*xyz* = [4, 33.5, -4], *t*_*19*_ = 4.77, *p* < 10^-4^, *k* = 83, SVC *p*_*FWE*_ < 0.05) (**Fig 7**) for the Good-learner group alone. Action-value signals were also identified bilaterally in vmPFC, albeit at an uncorrected threshold (**S4 Fig**).

### Poor-learner group

Focusing specifically on the Poor-learner group, the relevant fMRI effects were expected to be present to some extent but also weaker relative to the Good-learner group as a reflection of the less robust learning evident in behavior. In line with these expectations, SVPE and AVPE signals were only identified in the ventral striatum at an uncorrected threshold (**S5A and S5B Fig**). State-value signals were also found in vmPFC at an uncorrected threshold (**S5C Fig**).

### Good-learner group versus Poor-learner group

To an extent consistent with our initial hypothesis, direct contrasts between the Good-learner and Poor-learner groups with respect to both SVPE and AVPE signals revealed with uncorrected significance differences between the groups specifically in clusters within the dorsal striatum that overlap with those identified for the Good learners alone (**S6A and S6B Fig**). Another direct contrast with respect to action-value signals revealed a region of vmPFC overlapping with that identified as encoding action-value signals for the Good-learner group (**S6C Fig**), but this effect also did not reach corrected significance.

### Neuroimaging analysis based on the pure Q-learning model

Considering that the fits of the ACQ(λ) and Q(0) models to the behavioral data were comparable after formally penalizing model complexity, we conducted a separate fMRI analysis instead based on the Q(0) model and thus by design accounting for an AVPE signal alone rather than the AVPE together with the SVPE. The results for this AVPE were qualitatively similar to the results found for the AVPE derived from the ACQ model as reported above, and hence the Q(0) results are not reported in further detail here. Indeed, as with the AVPE signal initially produced by the ACQ model, no significant effects of the AVPE derived from the Q(0) model were found within the dopaminergic midbrain (*p* > 0.005).

## Discussion

Utilizing formal computational modeling together with high-resolution fMRI, we aimed to determine the nature of prediction-error signals encoded within dopaminergic nuclei of the tegmentum and efferent striatal structures during learning and performance of a sequential instrumental-conditioning task with an MDP including passive states. This novel task was designed to facilitate discrimination of two distinct forms of RPE signals—namely, the SVPE, by which errors in predictions about the expected values of successive states are used to update state values as well as action weights, and the AVPE, by which errors in predictions about the expected values of actions are used to update explicit action values. Furthermore, with multiple variants of RL algorithms to choose from such as the actor/critic model, action-value-learning models, and hybrid models, this approach enabled us to determine which variety of an RL model best accounts for not only behavior but also neural activity in the dopaminergic nuclei and their striatal targets during instrumental learning coupled with passive (i.e., Pavlovian) conditioning.

As a partial confirmation of our initial hypothesis and a contradiction to the assumptions of a strict action-value-learning model, we found evidence for the presence of SVPE signals within the dopaminergic midbrain—specifically, in the SN. Consistent with our expectations was evidence for an SVPE signal in the ventral striatum. On the other hand, contrary to what we initially expected, we also found evidence for SVPE signals in the caudate nucleus within a dorsal-striatal ROI previously implicated in instrumental conditioning [30,51].

The presence of SVPE signals in the dopaminergic midbrain as well as both the ventral striatum and the dorsal striatum provide direct evidence in support of the operation of an actor/critic mechanism in the basal ganglia [6,15–20]. According to this actor/critic theory, a common SVPE signal would be utilized by not only the ventral-striatal critic module to update a cached state value but also the dorsal-striatal actor module to update the action policy.

Our findings also suggest that the actor/critic dyad is not the only mechanism in play. A hitherto unexplored possibility was that learning here can be accounted for not by a pure actor/critic model alone nor even by an action-value-learning model alone but rather by a hybrid of the two models that combines predictions from their respective algorithms in order to compute net action weights. Complementing the SVPE signals within the striatum that would be produced by a state-value-learning algorithm, there was also distinct evidence for the representation of AVPE signals that would be produced by an action-value-learning algorithm. These AVPE signals were robustly represented within the ventral striatum alongside the SVPE signals described earlier. These AVPE signals also extended into the dorsal striatum [19], and there was evidence—albeit uncorrected—suggesting that dorsal-striatal AVPE signals were associated with superior learning performance on the task—being more strongly represented in Good learners than Poor learners. In harmony with the ACQ model, the findings of both the actor/critic model’s SVPE and the action-value-learning model’s AVPE within the striatum imply that both an actor/critic mechanism and an action-value-learning mechanism operate in parallel as part of an integrated learning system in the nigrostriatal circuit.

The evidence demonstrated here in support of the coexistence of two different computational strategies within the basal ganglia resonates with a burgeoning literature surrounding the notion of multiple learning and control systems that interact to collectively drive behavior [60,61]. Typically, such interactions have been suggested to take place between model-based control and model-free RL [54,62–64], as opposed to the interactions between two distinct model-free RL mechanisms emphasized here. In the present paradigm, we also sought possible evidence of model-based control or some hybrid of model-based and model-free learning. However, the results of our model comparison did not support significant involvement of a model-based system in the present experiment. This null result was likely for the reason that the MDP in the present study was not designed to elicit model-based control—being focused instead on dissociating the SVPE and the AVPE. Hence, model-free control was set up to be a sufficiently useful strategy for driving behavior on this task.

The present findings support the functioning of purely model-free actor/critic and action-value-learning mechanisms alongside each other but could possibly also align to some extent with other recent suggestions of roles for RL algorithms based on successor-state representations or latent-state representations in human learning [65–68]. Effectively occupying an intermediate position between the dichotomous extremes of model-free and model-based strategies, a successor-representation system constitutes a degenerate model-based system retaining some model-based features such as devaluation sensitivity without incurring the costly computational demands associated with encoding a rich model of the state space and explicitly computing action values via planning. Although the present task—having not been designed for such purposes—is not suited to assess evidence specifically in favor of a successor-representation scheme, there does remain a possibility that the action-value-learning component of our ACQ model in particular might be mimicking some effects of this more sophisticated system. In a similar vein, the “Dyna” architecture [69]—notwithstanding its less straightforward putative neural implementation—approximates model-based dynamic-programming methods but is also based on model-free action-value learning. Yet, additional work will be necessary to further dissociate and verify the predictions made by the different classes of models and hybrids of these across different experimental settings.

In addition to testing for signatures of prediction-error signals in the BOLD response, we also tested for signaling of the state values and action values being learned. We found evidence for each of these signals within vmPFC as expected. These findings align with previous reports of correlations with expected value for both actions and stimuli in this area [45,46,70]. However, the present findings do constitute an important advance beyond this previous literature in demonstrating the engagement of these two distinct value signals simultaneously during performance of a single integrated task. Furthermore, action-value signals in vmPFC were associated with superior performance of the task, whereas analogous state-value signals in vmPFC were not.

Another important feature of the present study that sets it apart from many previous studies of the representation of RL signals is the usage of a high-resolution functional-neuroimaging protocol. Along with optimized preprocessing and between-subject spatial normalization, this spatial resolution allowed us to discriminate not only signals in individual dopaminergic nuclei of the human midbrain but also signals at precise loci within the striatum. For instance, we were able to focally identify evidence for qualitatively distinct prediction-error signals within different subregions of the dorsal striatum. As such, the present study helps to provide new insights into potential specializations even within the dorsal subdivision of the striatum in terms of the computations encoded therein. Future high-resolution studies in turn can utilize our findings here as priors in order to motivate yet more specific hypotheses about regional specialization.

While the high-resolution protocol we used enables new insights into detailed functional neuroanatomy within nigrostriatal circuits, this approach is not without inherent technical challenges and limitations. Firstly, there are difficulties in applying techniques for multiple- comparison correction that were originally developed for conventional imaging protocols with lower resolution. This issue is not only due in part to the vastly increased (i.e., by roughly an order of magnitude) number of voxels that must be corrected for within a volume or a given region of interest but also perhaps to some extent due to the distributional (e.g., Gaussian) assumptions underpinning such multiple-comparison methods that might not apply in the same way for more finely sampled data. Another limitation of our high-resolution protocol is the tradeoff between resolution and signal-to-noise ratio in fMRI; as the voxel size is decreased, the signal-to-noise ratio decreases correspondingly. As a result of these challenges, only the results that we report in the main manuscript figures survived small-volume correction, whereas some of the other results reported (**S3–S6 Figs**) did not reach fully corrected significance within our a-priori search volumes. To ensure that these search volumes were as unbiased as possible, we used significant coordinates from the two meta-analyses on RL in the human brain that have been published to date [30,71]. However, as these meta-analyses were based on neuroimaging studies at conventional resolutions rather than the high spatial resolution available here, there was less potential to motivate more neuroanatomically precise hypotheses at this relatively early stage. This being exploratory research as such, we documented all of the effects that we found in the striatum—even for clusters that did not quite achieve corrected significance. These limitations notwithstanding, we have to note the important caveat that the uncorrected results reported in the supplementary figures will require further confirmation and should therefore be viewed as tentative. That said, these findings do in fact overlap sensibly with prior literature in expected ways, such as, for instance, the link we observed between not only AVPE-related activity but also SVPE-related activity in the dorsal striatum and behavioral performance, a trend that is consistent with and adds to previous findings by Schönberg and colleagues [51]. However, it remains possible that the between-group comparisons in the present study are somewhat underpowered, and thus larger sample sizes for the subgroups of Good learners and Poor learners would be warranted in a future study to confirm and further investigate the relationship between dorsal-striatal prediction-error signals and behavioral performance.

The contrast between the observed presence of SVPE signals in the SN and the absence of such significant effects in the VTA is also of note. Although one previous high-resolution fMRI study has reported parametric effects of RPE in the VTA as well as the SN [72], another study by our group identified RPE signals in the SN but not the VTA [38]. The absence of SVPE signals in the VTA could be a manifestation of the difficulty inherent to capturing BOLD responses related to prediction-error signals in this minute region [39–41] or instead might provide information about the specific roles (or lack thereof) for the VTA in a task of this variety. Another issue arising from the present findings is that while AVPE signals were observed in the striatum as expected, no such signals were found within the dopaminergic midbrain, which exclusively exhibited correlations with the SVPE. This discrepancy raises the question of how the AVPE signals in the striatum originate if correlates of these signals are not also evident in the dopaminergic midbrain. While it is important to avoid too strong of an inference from a null result—especially as a direct contrast between the SVPE and the AVPE did not reveal any significant differences—one possibility is that the AVPE is not computed within the dopaminergic nuclei at all. Rather, these AVPE signals may be computed elsewhere, whereby they serve to augment the information in the SVPE generated by a dopamine-mediated actor/critic system. A more prosaic explanation for this pattern could be that we have somewhat less statistical power to detect the AVPE as compared with the SVPE because the SVPE was elicited across both the passive and active states included in our MDP, whereas the AVPE was only present following active states in which participants actually performed an action and also had more of an opportunity to maximize reward and thus reduce signal variance. Yet, in spite of this difference, we nonetheless did observe robust AVPE signals throughout both the ventral striatum and the dorsal striatum while related effects were not present in the midbrain even at extremely lax statistical thresholds. These contrasting positive results suggest statistical power might not be the sole explanation for the observed difference in midbrain responsivity between the SVPE and the AVPE, but it will be important to follow up on these preliminary observations in order to reach more definitive conclusions about the role of the human dopaminergic midbrain in encoding of the AVPE or lack thereof.

To conclude, this study provides evidence that an actor/critic mechanism operating in concert with an additional action-value-learning mechanism provides an apt account of prediction-error-related neural activity within the human SN and the striatum. The SVPE was robustly encoded in the SN, the ventral striatum, and the dorsal striatum, which is consistent with the literal implementation of an actor/critic mechanism. On the other hand, we also observed evidence for signals related to the updating of action values per se, which is compatible with an additional integration of action-value learning into this architecture. Collectively, these results begin to shed light on the nature of the prediction-error computations emerging from the nigrostriatal system in the human brain.

## Materials and methods

### Ethics statement

Human participants provided informed written consent for protocols approved by the California Institute of Technology Institutional Review Board.

### Participants

Thirty-nine participants ranging between 18 and 39 years old from Caltech and the local community volunteered for the study. Participants were first screened for MRI contraindications. All participants were right-handed and generally in good health. Demographic information is included in **Table 1**. Participants were paid $40 for completing the study in addition to earnings from the task.

### Experimental procedures

Shown in **Fig 1** is a schematic of the task that includes transition probabilities for one of two Markov decision processes (MDPs) within one of three blocks as defined by said probabilities. A white fixation cross subtending 0.7° x 0.7° of visual angle was presented alone against the dark gray background throughout the intertrial interval (ITI). The duration of the ITI was drawn without replacement within a run from a discrete uniform distribution ranging from 4 to 8 s in increments of 80 ms. The fixation cross remained within the display at all times. Passive and active trial types and the two initial states specific to each occurred with equal probability. Trials were also ordered in a series of randomized quartets each including all four initial states for balance. A pre-trial cue with a duration of 1 s was first presented on either side of the fixation cross in the form of two white circles or two white arrows—for passive trials or active trials, respectively—each subtending 0.7° x 0.7° at an eccentricity of 2.4° to indicate an upcoming passive or active trial, respectively.

Following a pre-trial cue for a passive trial, one of two fractal cues subtending 3.7° x 3.7° that each represented a first-stage passive state appeared for 1.5 s with equal probability while the circles remained onscreen. The transition probabilities for the first-stage state determined which of two second-stage passive cues (i.e., fractals) was to be presented next for 1.5 s following an interstimulus interval (ISI) of 3.5 s. In consideration of the sensitivity of these learning algorithms to the timing of outcomes, the jitter typical of rapid event-related functional magnetic-resonance imaging (fMRI) studies was forgone here in favor of stable prediction-error signals. The transition probabilities for the second-stage state determined whether the final outcome reached after a second ISI of 3.5 s was an intact image of equal size depicting a dime, which with every encounter corresponded to an actual 10-cent reward, or a scrambled version of the coin’s image, which would correspond to the absence of reward for that trial. The scrambled version of the dime image was generated by dividing the intact image into an even 34 x 34 grid and randomly rearranging the resulting fragments.

Following a pre-trial cue for an active trial, one of two fractals that each represented a first-stage active state appeared with equal probability while the arrows remained onscreen. The subject was allotted 1.5 s to respond by pressing a button with either the left or the right index finger. Only the arrow corresponding to the subject’s choice continued to be displayed between the time of response and stimulus offset. The transition probabilities for the action given the state determined which of the aforementioned pair of second-stage passive states was to be presented next. Thus, passive and active trials were comparable in sequence and timing following offset of the first-stage cue. If the subject made a technical error by failing to respond in time for an active cue or responding inappropriately for a passive cue, only a red fixation cross was presented for the remainder of the trial as an indication of the loss of an opportunity to receive a reward.

The transition probabilities were structured with some degree of symmetry as follows. For a given block, a greater probability of transitioning to a reward state from one second-stage state would correspond to a lesser probability of reward for the other second-stage state. Likewise, a greater probability of transitioning to a given second-stage state from one first-stage passive state corresponds to a lesser probability of transitioning to that same second-stage state from the other first-stage passive state. The same inverse relationship applied to the action pairs for each of the active first-stage states, such that the mapping between actions and probabilities was inverted across the two states. To illustrate, if the left hand were to yield the greatest expected value for one active state, the right hand would yield the greatest expected value for the other active state. Optimal performance is therefore sharply defined in this context.

Prior to the main experiment, the subject was required to complete a 10-trial practice session during structural scanning with a distinct set of fractals and hypothetical monetary incentives. The subject was explicitly instructed in layperson’s terms that the trial sequence always retained the Markov property and did not maintain fixed transition probabilities across the course of the entire session. The 200 trials of the experiment were divided into a first block of 80 trials and two subsequent blocks of 60 trials each. The onset of a new block was defined by reversals of transition probabilities within an active state or between temporally aligned passive states. Although the subject was informed that the transition probabilities of the MDP could change throughout the session, no explicit indication of how or when reversals occurred was provided. Likewise, the onsets of each 50-trial scanning run were intentionally decoupled from the onsets of blocks. Factors counterbalanced together across subjects were based on whether the initial reversal occurred for the first stage or the second stage as well as the mapping of the arbitrarily defined actions to the left and right hands. This manipulation and the randomization of the sequences in each session overall ensured the generalizability of the observed effects when taking advantage of group-level analyses—but with the inevitable expense of added intersubject noise.

Stimuli were projected onto a 19-inch screen that was viewed in the MRI scanner with an angled mirror from a distance of 100 cm. The display was presented with a resolution of 1024 x 768 pixels and a refresh rate of 60 Hz. Fractal images were chromatic and had a resolution of 170 x 170 pixels. The mapping between the six fractal images and the states they represent was randomized for each subject. The interface was programmed using MATLAB and the Psychophysics Toolbox [73].

### Data acquisition

Magnetic-resonance imaging (MRI) data were acquired at the Caltech Brain Imaging Center using a 3-T Siemens Magnetom Tim Trio scanner and a 32-channel receive-only phased-array head coil. To guide the functional imaging, a structural volume of the entire brain was acquired first using a T1-weighted magnetization-prepared rapid gradient-echo (MPRAGE) sequence (repetition time (TR): 1500 ms, echo time (TE): 2.74 ms, inversion time (TI): 800 ms, flip angle (FA): 10°, voxel: 1.0 mm isotropic, field of view (FOV): 176 x 256 x 256 mm).

High-resolution functional images were acquired with a blood-oxygen-level-dependent (BOLD) contrast using a T2*-weighted gradient-echo echo-planar imaging (EPI) sequence (TR: 2770 ms, TE: 30 ms, FA: 81°, phase oversampling: 75%, acceleration factor: 2, voxel: 1.5 mm isotropic, FOV: 96 x 96 x 60 mm). The in-plane field of view of these images was restricted to covering the midbrain and the striatum using phase-encoding oversampling with controlled foldover. Forty contiguous slices were collected in interleaved-ascending order for each volume. Geometric distortions in EPI data were corrected using *B*_*0*_ field maps derived from dual gradient-echo sequences acquired between functional scanning runs (TR: 415 ms, TE_1_: 3.76 ms, TE_2_: 6.22 ms, FA: 60°, voxel: 2.5 x 2.5 x 2.6 mm, FOV: 200 x 200 x 125 mm). Cardiac and respiratory signals were recorded during scanning via a peripheral pulse oximeter and an abdominal bellows, respectively. The functional imaging was divided into four scanning runs, each having a duration of roughly 15 min that corresponded to 50 trials. The first two volumes of each run were discarded to allow for magnetization equilibration.

In the interest of discerning minute anatomical structures within the midbrain [50], the volumetric resolution of the functional pulse sequence (i.e., 3.4 mm^3^) was designed to be almost an order of magnitude lower than that achieved in more typical fMRI protocols with a standard isotropic spatial resolution between 3 mm and 4 mm corresponding to a volumetric resolution between 27 mm^3^ and 64 mm^3^. Such an enhancement could only be achieved at the expense of both the signal-to-noise ratio and the spatial extent of the functional images, leaving limited coverage beyond subcortical areas. Nevertheless, the reduced field of view did not interfere with the study inasmuch as its scope was to be restricted to the dopaminergic midbrain, the striatum, and ventromedial prefrontal cortex (vmPFC) a priori. Some omission of the rostralmost portion of vmPFC beyond the cingulate gyrus was tolerated because hypothetical value signals in vmPFC were assigned less priority than the hypothetical prediction-error signals in the basal ganglia that form the cornerstone of the present research.

### The actor/critic (AC) model

Considering algorithms for reinforcement learning (RL) [1–3] via the temporal-difference (TD) prediction method [5], the first candidate for model-free (i.e., habitual) learning [21,22] was the actor/critic (AC) model [12–14]. The AC model posits that the only reward-prediction error (RPE) that is computed is a state-value-prediction error (SVPE). The “critic” module central to this feedback-driven learning process lacks any representation of actions despite transmitting common input to the “actor” module. Thus, the algorithm is simpler and somewhat more parsimonious than the action-value-learning algorithm detailed below in spite of comparable free parameters.

The RL framework reduces the environment to an MDP in terms of sets of states *s ∊ S* and actions *a ∊ {A|s}*. Considering that the novel cues have no previous associations with reinforcers, a naïve agent lacks priors for value estimates and therefore initializes the expected values of these states *V*_*t*_*(s)* to zero:

$$\forall \;s:\;V_{0}(s)=0$$

For each state transition within a trial, the TD algorithm updates the previous state-value estimate *V*_*t*_*(s*_*t*_*)* by computing the SVPE *δ*^*V*^_*t*_ as determined by either the current reward *r*_*t+1*_ or the current value estimate *V*_*t*_*(s*_*t+1*_*)* predicting future rewards or lack thereof. The standard discount factor *γ* was omitted here (i.e., *γ* = 1) inasmuch as only one reward could be delivered after a constant delay across all trials, leaving this reduced delta-learning rule:

$$\delta_{t}^{V}=r_{t+1}+V_{t}(s_{t+1})-V_{t}(s_{t})$$

This model is formally referred to as the “AC(λ)” model with the addition of the “TD(λ)” eligibility trace that facilitates rapid learning across serial events. The eligibility trace of this TD(λ) prediction-error signal weights updates prior to the most immediate one according to the eligibility *λ* as the base of an exponential function modulating the learning rate *α*. With discretely episodic paradigms such as in the present study, the eligibility trace only propagates back to trial onset *t*_*0*_. Thus, for *λ* > 0, the final state transition within a trial not only updates the value estimate for the second-stage cue by *αδ*^*V*^_*t*_ but also updates the value estimate for the first-stage cue by *αλδ*^*V*^_*t*_ as follows (where Ζ* denotes the set of nonnegative integers):

$$\forall \;\{n\in Z^{*}\;|\;n\leq t-t_{0}\}:\;V_{t+1}(s_{t-n})=V_{t}(s_{t-n})+\alpha \lambda^{n}\delta_{t}^{V}$$

Rather than representing the expected values of individual actions in the AC model, the actor of the actor/critic dyad encodes the weights of its stochastic action-selection policy *π*_*t*_*(s*,*a)* in proportion to relative action preferences *p*_*t*_*(s*,*a)* that are likewise initialized to zero and then updated by the same SVPE *δ*^*V*^_*t*_:

$$\forall \;\{n\in Z^{*}\;|\;n\leq t-t_{0}\}:\;p_{t+1}(s_{t-n},a_{t-n})=p_{t}(s_{t-n},a_{t-n})+\alpha \lambda^{n}\delta_{t}^{V}$$

### The Q-learning (Q) model

Representing in contrast the action-value-learning methods, the Q-learning (Q) model [32] remains within the domain of model-free RL but takes the slightly more efficient approach of computing action values for active states and utilizing an action-value-prediction error (AVPE) in doing so. In its purest form, the Q model lacks representations of state-value estimates and thus is insensitive to passive states as conditioned reinforcers. In lieu of the state values characteristic of the AC model, the action values *Q*_*t*_*(s*,*a)* are initialized to zero:

$$\forall \;(s,a):\;Q_{0}(s,a)=0$$

The Q model’s more complex variant of the TD algorithm updates the previous action-value estimate *Q*_*t*_*(s*_*t*_,*a*_*t*_*)* by computing the AVPE *δ*^*Q*^_*t*_ as determined by either the current reward *r*_*t+1*_ or the maximum of the current action-value estimates *Q*_*t*_*(s*_*t+1*_,*a)* predicting rewards or lack thereof:

$$\delta_{t}^{Q}=r_{t+1}+\underset{a^{\prime }}{\max}Q_{t}(s_{t+1},a^{\prime })-Q_{t}(s_{t},a_{t})$$

Again, the TD(λ) prediction-error signal would generate an eligibility trace that extends backward in time beyond the most recent state and action:

$$\forall \;\{n\in Z^{*}\;|\;n\leq t-t_{0}\}:\;Q_{t+1}(s_{t-n},a_{t-n})=Q_{t}(s_{t-n},a_{t-n})+\alpha \lambda^{n}\delta_{t}^{Q}$$

However, in the case of the present study, there was only a single action available per episode, meaning that only the Q(0) model lacking an eligibility parameter *λ* could be fitted. A related aspect of the present paradigm was that the “off-policy” Q-learning method could not be readily distinguished from an “on-policy” counterpart such as the state-action-reward-state-action (SARSA) method [33]. The former computes an AVPE using the maximal expected value across subsequently available actions, whereas the latter computes an AVPE using the value of the action actually chosen according to the current policy. For clarity in this study, we elected to focus on only Q learning as the canonical archetype of an action-value-learning algorithm and thus do not consider the SARSA model further.

### The critic/Q-learner (CQ) model

The hybridized “critic/Q-learner” (CQ) model essentially retains the action values and the AVPE of the Q model for active states but also represents both active and passive states in terms of state values and the SVPE as the critic would even in the absence of its complementary actor. If active and passive states are in sequence as in the present study, more information becomes available to guide control for the CQ model than for a pure action-value-learning model.

To adhere to the equations described in the preceding sections, the values of passive states *V*_*t*_*(s)* and the values of state-action pairs *Q*_*t*_*(s*,*a)* can nominally be referred to collectively for the CQ model with the introduction of a null “pseudoaction” *A_0_*. However, this notational simplification should not be misconstrued as implying actual equivalence in the neural representations of the SVPE *δ*^*V*^_*t*_ and the AVPE *δ*^*Q*^_*t*_, which still function separately for passive and active states, respectively:

$$\forall \;\{s\;|\;\{A\;|\;s\}=\emptyset \}:\;Q_{t}(s,A_{0})\equiv V_{t}(s)$$

### The actor/critic/Q-learner (ACQ) model

Although the actor/critic and Q-learning models have typically each been considered in isolation, they are neither mutually exclusive in practice nor mutually exclusive in theory. The “actor/critic/Q-learner” (ACQ) model was introduced as a novel model-free hybrid that incorporates the SVPE as well as the AVPE into updates for active states according to a parameter for action-value weighting, *w*_*Q*_. The AC model (i.e., *w*_*Q*_ = 0) and the CQ model (*w*_*Q*_ = 1) are thus both nested in the ACQ model. Such hybridization entails the representation of net action values *Q*^*V*^_*t*_*(s*,*a)* incorporating both action and state values. One possible interpretation of this integration could be that the simpler (but also more generalizable) information maintained within the critic module leaks into the richer action-specific representations of value within the Q-learner module:

$$Q_{t}^{V}(s,a)=w_{Q}Q_{t}(s,a)+(1-w_{Q})V_{t}(s)$$

The complete ACQ(λ) model retains not only the SVPE and the AVPE but also the respective eligibility traces for each of the dual updates as described in the preceding models. The weighting parameter *w*_*Q*_ likewise dictates the net action-value-prediction error *δ*^*Q*,*V*^_*t*_ as follows:

$$\delta_{t}^{Q,V}=w_{Q}\delta_{t}^{Q}+(1-w_{Q})\delta_{t}^{V}$$

The ACQ model does not directly factor net action values into the decision-making process, however. Rather, the SVPE *δ*^*V*^_*t*_ and the AVPE *δ*^*Q*^_*t*_ similarly update a net action weight *W*_*t*_*(s*,*a)* that integrates the actor’s action preference *p*_*t*_*(s*,*a)* and the Q-learner’s action value *Q*_*t*_*(s*,*a)* as combined inputs to the policy *π*_*t*_*(s*,*a)*:

$$W_{t}(s,a)=w_{Q}Q_{t}(s,a)+(1-w_{Q})p_{t}(s,a)$$

### The model-based (MB) model

As model-free learning was the primary focus of the present study, the task was not designed in such a way that a model-based (i.e., goal-directed) learning [52] system would be likely to take effect. Nevertheless, only a rigorous model comparison as conducted here could entirely rule out the possibility of more complex model-based learning as opposed to direct RL.

The model-based (MB) model [3,53,54] features an optimal dynamic-programming algorithm that—unlike the TD algorithm—plans forward in time and maintains explicit estimates of the transition probabilities of the MDP as part of a transition function *T*. Diverging from the model-free learner’s estimates of value even at the first time step, a naïve model-based learner initializes the transition matrix with uniform priors over feasible target states *s’ ∊ {S|(s*,*a)}*, which happen to always be binarized in this case. Adhering to the convention used for Q-learning, passive and active states are not differentiated merely for the sake of readability:

$$\forall \;(s,a,s^{\prime }):\;T_{0}(s,a,s^{\prime })=1/|\{S\;|\;(s,a)\}|=1/2$$

The MB algorithm updates the probability estimates by computing a state-prediction error (SPE) δ*_t_ analogous to the model-free RPE (i.e., the SVPE *δ*^*V*^_*t*_ or the AVPE *δ*^*Q*^_*t*_) but unique in that is determined by the probability of the outcome state *s*_*t+1*_ itself:

$$\delta_{t}^{*}=1-T_{t}(s_{t},a_{t},s_{t+1})$$

The estimated probability of the observed transition is thus increased in accordance with the model-based learning rate α*:

$$T_{t+1}(s_{t},a_{t},s_{t+1})=T_{t}(s_{t},a_{t},s_{t+1})+\alpha^{*}\delta_{t}^{*}$$

The probability estimates for all transitions other than that observed must be proportionally decreased as well:

$$\forall \;\{s^{\prime }\;|\;(s_{t},a_{t})\wedge s^{\prime }\neq s_{t+1}\}:\;T_{t+1}(s_{t},a_{t},s^{\prime })=T_{t}(s_{t},a_{t},s^{\prime })-\alpha^{*}T_{t}(s_{t},a_{t},s^{\prime })$$

Utilizing the transition function, the MB learner’s action-value estimates Q*_t_*(s*,*a)* correspond to explicit expectations for successor states, their outcomes in turn, and their known rewards per a reward function *R(s)*. Whereas model-free value estimates at the first stage are updated only on trials for which they have been encountered, all of their model-based counterparts are updated on every trial with the influx of any new information. The dynamic-programming algorithm accomplishes this by recursively evaluating the following Bellman optimality equation:

$$\forall \;(s,a):\;Q_{t+1}^{*}(s,a)=\sum_{s^{\prime }\in S|(s,a)}T_{t+1}(s,a,s^{\prime })\left(R(s^{\prime })+\underset{a^{\prime }}{\max}Q_{t+1}^{*}(s^{\prime },a^{\prime })\right)$$

### Computational modeling of action selection

The “ACQ(λ)+MB” model, which is the full hybrid model within which every reduced model was nested, assumes that both model-free systems and the model-based system all operate as subcomponents in parallel. As the ACQ model already specifies a net action weight *W*_*t*_*(s*,*a)* for model-free learning, the model-based weighting parameter w* controls the weighting of model-based input relative to model-free and thus accommodates the cases of exclusively model-free learning (i.e., w* = 0), exclusively model-based learning (w* = 1), or both types of learning in parallel (0 < w* < 1) with a model-based/model-free net action weight W*_t_*(s*,*a)*:

$$W_{t}^{*}(s,a)=w^{*}Q_{t}^{*}(s,a)+(1-w^{*})W_{t}(s,a)$$

With regard to action selection, all of the learning algorithms converge on a Gibbs softmax model [3,74,75]. This augmented version models hysteresis via a perseveration bias *β*_*t*_*(s*,*a)* [58] as well as a constant choice bias *β*_*R*_ with the arbitrary convention that positive and negative map onto rightward and leftward biases, respectively. Learned and intrinsic biases were all incorporated into the probabilistic action-selection policy *π*_*t*_*(s*,*a)* via the following softmax function with temperature *τ*, which regulates the stochasticity of choices. This equation reduces to a logistic function in this paradigm’s two-alternative forced-choice task:

$$\pi_{t}(s_{t},a)=P(a_{t}=a\;|\;s_{t})=\frac{\exp\left\{(W_{t}^{*}(s_{t},a)+\beta_{t}(s_{t},a)+\beta_{R}I_{R}(a))/\tau \right\}}{\sum_{a^{\prime }\in A|s_{t}}\exp\left\{(W_{t}^{*}(s_{t},a^{\prime })+\beta_{t}(s_{t},a^{\prime })+\beta_{R}I_{R}(a^{\prime }))/\tau \right\}}$$

Modeling hysteresis in terms of the dynamics of cumulative perseveration biases first requires an initialization of *β*_*t*_*(s*,*a)*, which is here notated so as not to be confused with the parameter *β*_*0*_:

$$\forall \;(s,a):\;\beta_{t=0}(s,a)=0$$

A counter variable *C*_*t*_*(s)*, indexing the number of arrivals to a state *s*, is similarly initialized:

$$\forall \;s:\;C_{0}(s)=0$$

The arrival-counter variable is simply incremented after each encounter with a given state:

$$C_{t}(s_{t})=C_{t-1}(s_{t})+1$$

According to this arrival index, the indicator function *I*_*C(s)*_*(s*,*a)* tracks the history of all state-action pairs:

$$\forall \;\{a\;|\;s_{t}\}:\;I_{C_{t}(s_{t})}(s_{t},a)=\left\{ \begin{array}{c} \begin{array}{cc} \;1, & a=a_{t} \end{array}\\ \begin{array}{cc} \;0, & a\neq a_{t} \end{array} \end{array}\right.$$

The exponential decay of the perseveration bias is determined by its initial magnitude *β*_*0*_ and inverse decay rate *λ*_*β*_. The latter is notated with the convention used for the eligibility trace, such that *λ* and *λ*_*β*_ both correspond to the complement of (i.e., unity minus) the decay rate. The exponential decay of a perseveration bias occurs within a state per each action executed in that state, as described in the following equation that integrates cumulative perseveration biases:

$$\forall \;\{a\;|\;s_{t}\}:\;\beta_{t+1}(s_{t},a)=\sum_{n=0}^{C_{t}(s_{t})-1}\beta_{0}\lambda_{\beta }^{n}I_{C_{t}(s_{t})-n}(s_{t},a)$$

Finally, the indicator function *I*_*R*_*(a)* arbitrarily dictates the constant choice bias like so (where “R” and “L” stand for right action and left action, respectively):

$$I_{R}(a)=\left\{ \begin{array}{c} \begin{array}{cc} \;1, & a=A_{R} \end{array}\\ \begin{array}{cc} \;0, & a=A_{L} \end{array} \end{array}\right.$$

The full ACQ(λ)+MB model includes nine free parameters altogether—viz., model-free learning rate *α*, eligibility *λ*, action-value weight *w*_*Q*_, model-based learning rate α*, model-based weight w*, softmax temperature *τ*, rightward bias *β*_*R*_, and initial magnitude *β*_*0*_ coupled with inverse decay rate *λ*_*β*_ for exponential decay of the perseveration bias—with the following constraints: 0 ≤ *α* ≤ 1, 0 ≤ *λ* ≤ 1, 0 ≤ *w*_*Q*_ ≤ 1, 0 ≤ α* ≤ 1, 0 ≤ w* ≤ 1, *τ* > 0, 0 ≤ *λ*_*β*_ ≤ 1. The different types of model-free learning, eligibility traces either decaying or constant (i.e., *λ* = 1), and model-based learning were all counterbalanced factors in the formal comparison of 22 nested models.

### Model fitting

Along with the hysteresis model and a null intercept model, 21 learning models—namely, Q(0), AC(0), AC(1), AC(λ), CQ(0), CQ(1), CQ(λ), ACQ(0), ACQ(1), ACQ(λ), MB, Q(0)+MB, AC(0)+MB, AC(1)+MB, AC(λ)+MB, CQ(0)+MB, CQ(1)+MB, CQ(λ)+MB, ACQ(0)+MB, ACQ(1)+MB, and ACQ(λ)+MB—were all fitted to each individual subject’s behavior using maximum likelihood estimation. By capturing constant choice biases and response perseveration or alternation, the 4-parameter hysteresis model with learning rates fixed at zero offers a nested learning-independent control model more viable than the null intercept model with its lone parameter *P(A*_*1*_*)*. Thus, sensitivity to outcomes or lack thereof can be detected with greater precision by setting the performance of the hysteresis model as a benchmark for comparison with learning models. Tuning parameters were optimized with respect to goodness of fit for each subject using iterations of the Nelder-Mead simplex algorithm [76] with randomized seeding.

To adjust for model complexity when performing the model comparisons, we used the Akaike information criterion with correction for finite sample size (AICc) [55,56]. The preferred model ideally balancing parsimony and accuracy on the basis of the behavioral model fits would then be used for the subsequent neuroimaging analysis. To verify the discriminability of the preferred ACQ(λ) model here, each fitted instantiation of the model was used to simulate an artificial data set yoked to that of the respective subject for another model comparison. Furthermore, an artificial data set was also simulated in accordance with the ACQ model, and the same model comparison was conducted for that data set to verify that the ACQ model could in principle be discriminated among the alternatives here (**S1 Fig**).

### Data analysis: Behavior

Performance on the learning task was assessed for each subject by calculating overall accuracy—that is, the proportion of choices for which the subject chose the option more likely to ultimately result in delivery of an actual reward. The earliest trials in which the subject encounters a state for the first time and thus lacks information were excluded from this metric. Accuracy was compared with the chance level of 50% for each subject using a one-tailed binomial test. Subjects were initially divided into the “Good-Learner” and “Poor-learner” groups a priori according to whether or not accuracy was significantly greater than the chance level. The “Nonperformer” group was subsequently distinguished as the subset of Poor learners whose behavior is best accounted for by the hysteresis model. As the hysteresis model is characterized by absolute insensitivity to outcomes, Nonperformer subjects were necessarily excluded from further analysis.

Accuracy was compared with the chance level across subjects within the Good-learner group and within the Poor-learner group using one-tailed one-sample *t* tests. Accuracy was compared between subject groups using a two-tailed independent-samples *t* test. Similarly tested for between groups were possible confounds in the form of differences in reaction time (RT), errors such as missed or inappropriate responses that resulted in missed trials, or the demographic variables of age and gender. Utilizing the fitted parameters for each subject, the sensitivity of each instantiation of the ACQ(λ) model, which was preferred by the AICc, was calculated as *log(α(1+λ)/τ)*. With logarithmic transformation of this metric, zero sensitivity corresponds to a balance between the eligibility-adjusted learning rate and the temperature; absolute insensitivity to outcomes instead produces a sensitivity score approaching negative infinity. Positive sensitivity was tested for across subjects within each group using one-tailed one-sample *t* tests. Sensitivity was compared between groups using a one-tailed independent-samples *t* test, and post-hoc tests were subsequently conducted for learning rate, eligibility, and temperature. Finally, a positive correlation between model sensitivity and empirical choice accuracy was tested for across all subjects using linear regression and a one-tailed one-sample *t* test.

Taking quantitative estimates of internal signals as predicted by the fitted models, subjects’ choices were analyzed with two complementary logistic-regression models. The first modeled the probability of a right-action choice *P(a*_*t*_ *= A*_*R*_*)* as a function of the difference between the right and left options’ net action weights *W*_*t*_*(s*_*t*_,*A*_*R*_*)* and *W*_*t*_*(s*_*t*_,*A*_*L*_*)*. The second modeled the probability of a “stay” choice as a function of the difference between the net “stay” and “switch” weights, where “staying” or “switching” in this context refer to repeating the previous action given the current state or instead switching to another action, respectively. Subjects’ RTs were analyzed with a linear-regression model that captured the RT as a function of the absolute value of the difference between the right and left net action weights. In order to accommodate intersubject variability in the range of estimated values encountered throughout a session, these differences in net action weights were normalized with respect to the maximum absolute value for each subject. In preparation for the aggregate RT analysis, excessively fast contaminant observations were omitted at a threshold of 300 ms, which accounts for the cumulative temporal constraints of visual recognition, decision making, and motoric execution. Parameters for these mixed-effects models were first estimated at the level of an individual subject and assessed using one-tailed one-sample *t* tests. Parameter estimation was conducted using MATLAB and the Statistics and Machine Learning Toolbox. Choice curves were plotted with inner bins having width equal to 0.2 times the maximum weight difference and bins at the edges having width equal to 0.3 times the maximum.

### Data preprocessing

Preprocessing of neuroimaging data was mostly conducted using the FMRIB Software Library (FSL) (Centre for Functional MRI of the Brain, University of Oxford). Preprocessing steps included unwarping with field maps, slice-timing correction, motion correction, and high-pass temporal filtering at 0.01 Hz.

Denoising of data first required spatial independent-component analysis (ICA), which was implemented via the MELODIC (multivariate exploratory linear optimized decomposition into independent components) routine [77] in FSL. Following decomposition, artifactual noise components were identified and removed using the FIX denoising algorithm [78] in FSL. Moreover, the time courses of the five ICA components ranked with the greatest weights in the interpeduncular cistern were extracted for subsequent inclusion as regressors of no interest in the general linear model (GLM) (as in [38,79]). In addition to suffering an already poor signal-to-noise ratio, BOLD signals from the brainstem are especially susceptible to physiological artifacts [42–44], and the proximity of the pulsatile interpeduncular cistern to the tegmentum warranted this additional direct approach. Yet another solution to physiological contamination lay in modeling actual cardiac and respiratory signals with the RETROICOR (retrospective image correction) method [80] as carried out by the Physiological Log Extraction for Modeling (PhLEM) Toolbox [81] with bandpass filters. High- and low-frequency phase information was extracted along with the broadband photoplethysmogram; the respective time courses were all to be included as regressors of no interest. Fourier decomposition was also utilized for respiration before incorporating its time course into the GLM as regressors of no interest.

Functional images were coregistered to a high-resolution (i.e., 0.7-mm isotropic), multimodal template [82] in Montreal Neurological Institute (MNI) space with nearest-neighbor interpolation using the Advanced Normalization Toolbox (ANTs) [83]. All coordinates are accordingly reported in MNI space. This template is multimodal (or multivariate) in the sense of integrating complementary information from both T1 weighting and T2 weighting, thus enabling more precise alignment and delineation of subcortical structures and the brainstem in particular. The final step was spatial smoothing via an isotropic Gaussian kernel with a full width at half maximum (FWHM) of 2 mm, which was reduced from the standard 8-mm FWHM to preserve the fine granularity critical for detecting mesencephalic signals [30].

### Data analysis: Neuroimaging

Analysis of fMRI data was conducted using Statistical Parametric Mapping (SPM) (Wellcome Trust Centre for Neuroimaging, University College London). The computational-model-based analysis [59] utilized the ACQ(λ) model with subject-specific parameters as fitted for each individual. The GLM of BOLD signals was essentially characterized by four parametric regressors derived from the ACQ(λ) model—SVPE *δ*^*V*^_*t*_, state value *V*_*t*_*(s*_*t*_*)*, AVPE *δ*^*Q*^_*t*_, and action value *Q*_*t*_*(s*_*t*_,*a*_*t*_*)*. These corresponded to four indicator variables as boxcar functions each with their own respective parametric modulators. Action-value and AVPE signals were assumed to occur during and immediately following (i.e., after the ISI) active states, respectively. An active state was defined as one in which the subject was to select an action in order to proceed to the subsequent state. The intermediate state that immediately followed an active state was incorporated into the AVPE computation because the updates of TD algorithms require comparison of successive value predictions in two temporally adjacent states in this context. State-value and SVPE signals were assumed to occur during and immediately following both active and passive states. A passive state was defined as one during which no action was required on behalf of the agent in order to transition to the subsequent state. Also included in the analysis were the ITI and the pre-trial cues (i.e., those cues indicating which type of trial was coming) coded as passive states with concomitant state-value and SVPE signals in a manner similar to those of all of the states denoted by the fractal images. The duration of each boxcar function corresponded to the duration that a particular stimulus was presented with the exception that expected-value signals were also assumed to persist beyond stimulus offset through a subsequent ISI on the grounds that one’s expectations should remain the same during this interval between relevant states. Positive and negative prediction errors were represented symmetrically about zero along a common linear scale. To better account for signal variance overall, additional indicator variables in the form of boxcar functions lacking parametric modulators were used to define the onset of various events within the sequence of a trial—specifically, the passive-trial cue, the active-trial cue, the passive states with fractals, active states for choices of the left action, active states for choices of the right action, rewarded or unrewarded outcome states, and the onset of the fixation cross during both ISIs and ITIs. Moreover, events were included as separate regressors for trials during which an error such as a missed response or an inappropriate response occurred and prematurely ended the trial.

To rule out the possibility of signals that are in actuality AVPE signals contaminating the SVPE signal, the AVPE was extended to include error signals that updated a post-action state value but also could update the preceding action’s weight via an eligibility trace. Although AVPE signals overlap in time with the SVPE signals that correspond to the values of active states, the SVPE regressors also extending throughout passive states were clearly dissociable from the AVPE regressors by this design (mean *r* = 0.570). This multicollinearity was sufficiently subtle for the regression to not require an orthogonalization procedure that could potentially distort the results or their interpretation [84].

All of the above predictor variables were convolved with a canonical double-gamma hemodynamic-response function. We also included as nonconvolved regressors 6 movement parameters (i.e., 3 translation and 3 rotation), 2 variables for respiration, 9 variables for blood circulation (i.e., 4 high-frequency, 4 low-frequency, and 1 broadband), 5 ICA components from the interpeduncular cistern, a first-degree autoregressive (i.e., “AR(1)”) term, and a constant term. GLMs were first estimated at the level of an individual subject, and contrasts of parameter estimates were subsequently computed for the parametric regressors at the group level as part of a mixed-effects analysis. Positive effects of these contrasts were tested for using one-tailed one-sample *t* tests. The Good-learner and Poor-learner groups were analyzed collectively as well as separately for juxtaposition. Furthermore, direct contrasts of the Good-learner and Poor-learner groups with respect to these parametric effects were tested in an independent voxel-wise manner using one-tailed independent-samples *t* tests.

A pair of recent meta-analytical studies—the only two such studies to date—were consulted to constrain the hypothesis space, as their findings encompass various fMRI results for RPE signals. These studies are henceforth referred to as “GED” [71] and “CKED” [30]. The default thresholds for statistical significance and cluster extent were preset at standard levels of *p* < 0.005 and *k* ≥ 10 voxels [85,86]. Whole-brain correction was precluded by so many voxels being sampled with high resolution. Regardless of this, coordinates from the meta-analyses could guide a-priori regions of interest (ROIs) as part of small-volume correction (SVC) for multiple comparisons controlling the familywise error (FWE) rate at the cluster level. ROIs were defined for the dopaminergic midbrain, the ventral striatum, the dorsal striatum, and vmPFC as spheres with 7.5-mm radii centered at loci derived from rounded averages of two estimates offered by the meta-analyses, which were mostly in agreement.

The first two ROIs were defined by virtue of their association with RPE signals in appetitive Pavlovian and instrumental conditioning. The ROI for the dopaminergic midbrain was centered on the left side at (*x* = -9.5, *y* = -20.5, *z* = -10), taken from GED’s and CKED’s local maxima at (-10, -20, -8) and (-10, -20, -6), respectively, after rounding and with a minor 3-mm ventral translation to better align with the precise location of this structure in the anatomical template used. The ROI for the ventral striatum was defined bilaterally near the boundary of the ventral putamen and the nucleus accumbens with noncontiguous centers at (14.5, 6.5, -8.5) and (-14, 6.5, -8.5), taken from the average of GED’s and CKED’s peaks at (-10, 6, -6) and (-20, 6, -12), respectively. An ROI for the dorsal striatum was defined in the left caudate nucleus at (-9.5, 6.5, 14), taken from GED’s and CKED’s maxima at (-8, 4, 18) and (-10, 8, 10), respectively, for putative contrasts of instrumental as opposed to Pavlovian conditioning. Finally, only one meta-analysis furnished predictions for the ROI in vmPFC, which has been associated with expected value in RL paradigms; hence, a bilateral ROI centered at (-0.5, 30.5, -13) extracted both of CEKD’s peaks at (4, 34, -6) and (-6, 28, -20). SVC is reported for all clusters that were identified in whole-brain analyses and additionally withstood correction within these ROIs.

Furthermore, the high spatial resolution of both anatomical and functional images allowed for activity in the dopaminergic midbrain to be localized more specifically to either the VTA or the SN [50]. The tissue contrast revealed with T2-weighted structural images is particularly informative inasmuch as the SN and the red nucleus have distinctively low intensity in these images and mark boundaries of the VTA with its conspicuously greater intensity.

## Supporting information

S1 Fig Model discriminability. The model comparison reported in **Fig 2A** was replicated using artificial data that were simulated with the ACQ(λ) model as fitted for each subject but otherwise yoked to the empirical data set. Average goodness of fit relative to the outcome-insensitive hysteresis model across performing subjects is shown for each model tested with (light bars) and without (light and dark bars combined) a penalty for model complexity according to the AICc. A positive residual corresponds to a superior fit. As expected, only the ACQ(λ)+MB model—within which the actual model is nested—surpassed the actual model with respect to raw goodness of fit, but this overfitting was fully neutralized after correcting for model complexity. Degrees of freedom are listed in parentheses.
(TIF)

S2 Fig Model predictions. Representative dynamics of value signals and learning signals as generated by the ACQ(λ) model are Illustrated with the final subject from the Good-learner group. Fitted parameters were assigned as follows for this subject: *α* = 0.639, *λ* = 0.322, *w*_*Q*_ = 0.857, *τ* = 0.197, *β*_*0*_ = -0.046, *λ*_*β*_ = 0.976, and *β*_*R*_ = 0.193. **(a-b)** The model’s estimates (solid lines) of state value (SV) *V*_*t*_*(s)* as the probability of reward for the active states independent of actions are displayed in the upper-left corners of each panel along with empirical values (dashed lines) over the course of the experiment. Displayed in the upper-right corners are the state-value-prediction error (SVPE) *δ*^*V*^_*t*_ signals that for active states update not only the critic module’s state values *V*_*t*_*(s)* but also the actor module’s relative action preferences *p*_*t*_*(s*,*a)*, which are shown in the lower-left corners of each panel. As derived from the Q-learning component of the model, estimates of action value (AV) *Q*_*t*_*(s*,*a)* for the left and right options (red and green, respectively) are plotted at the left side of each panel along with empirical values. Each colored circle indicates an occurrence of the respective action. Adjacent to these plots on the right side of each panel are the time courses of the action-value-prediction error (AVPE) *δ*^*Q*^_*t*_ signals updating the action values. Net action weights *W*_*t*_*(s*,*a)* that integrate the aforementioned action preferences and action values are shown in the lower-right corners of each panel. **(c-d)** Time courses of state values and the SVPE are plotted for the first-stage passive states. **(e-f)** As plotted here, the SVPE for the second-stage passive states additionally updated representations for the first-stage states and actions via the eligibility trace. For this subject, a probability reversal at the second stage occurred before a probability reversal at the first stage.
(TIF)

S3 Fig Action-value-prediction-error signals. **(a)** For the Good-learner group, AVPE signals were identified throughout both the ventral striatum and the dorsal striatum. As with the aggregate analysis, the global peak of a cluster also within the ROI for the right ventral striatum (*xyz* = [8.5, 11, -2.5], *t*_*19*_ = 4.02, *p* < 10^-3^, *k* = 71, SVC *p*_*FWE*_ = 0.064) was actually located in the dorsal striatum (*xyz* = [11.5, 20, -2.5], *t*_*19*_ = 4.13, *p* < 10^-3^). The corresponding anterior-caudate region in the left hemisphere (*xyz* = [-8, 18.5, -7], *t*_*19*_ = 3.53, *p* = 10^-3^, *k* = 14) was likewise engaged in this way. The anterior-caudate regions identified here are in close proximity to those reported for an instrumental RPE signal by O’Doherty and colleagues [19], both falling within 7.5 mm of the previously reported peak and its mirror-symmetric location. More caudally, AVPE signals were also observed in the right dorsal putamen (*xyz* = [28, 6.5, -1], *t*_*19*_ = 3.30, *p* = 0.002, *k* = 17). The last of these clusters distinguished the Good-learner and Poor-learner groups (**S6B Fig**) and was to be found in the left dorsal striatum (*xyz* = [-20, 11, 0.5], *t*_*19*_ = 4.12, *p* < 10^-3^, *k* = 58) for the most part but also extended somewhat into the ventral striatum. Otherwise, these results mostly aligned with those of the aggregate analysis of Good learners and Poor learners together. **(b)** Across all of these performing subjects, there were corrected significant results in the ventral striatum in both the left (*xyz* = [-12.5, 11, -5.5], *t*_*34*_ = 4.44, *p* < 10^-4^, *k* = 115, SVC *p*_*FWE*_ < 0.05) and the right (*xyz* = [8.5, 12.5, -4], *t*_*34*_ = 3.87, *p* < 10^-3^, *k* = 108, SVC *p*_*FWE*_ < 0.05) hemispheres as previously mentioned. Despite having local maxima within the ventral striatum, however, these same clusters also extended into regions of the dorsal striatum outside of the primary ROI with global peaks elsewhere in both the left (*xyz* = [-20, 11, -2.5], *t*_*34*_ = 4.55, *p* < 10^-4^) and the right (*xyz* = [11.5, 20, -2.5], *t*_*34*_ = 4.24, *p* < 10^-4^) hemispheres.
(TIF)

S4 Fig Good-learner group: Action-value signals. In addition to the separate types of RPE signals, separate types of value signals were evoked by the current paradigm. Among the Good-learner group, action-value signals were identified bilaterally in vmPFC (*xyz* = [1, 33.5, -17.5], *t*_*19*_ = 3.87, *p* < 10^-3^, *k* = 21, SVC *p*_*FWE*_ = 0.086) as anticipated with marginal corrected significance.
(TIF)

S5 Fig Poor-learner group. **(a)** For the Poor-learner group, the relevant neural signals were expected to be weaker as a reflection of the less robust learning evident in behavior. In line with this expectation, SVPE signals were only identified in the right ventral striatum (*xyz* = [19, 11, -11.5], *t*_*14*_ = 4.92, *p* = 10^-4^, *k* = 13). **(b)** Correspondingly, AVPE signals were limited to the left ventral striatum (*xyz* = [-12.5, 9.5, -5.5], *t*_*14*_ = 4.64, *p* < 10^-3^, *k* = 44, SVC *p*_*FWE*_ = 0.056) among the Poor learners. **(c)** Although action-value signals were not observed in vmPFC at this statistical threshold for the Poor-learner group as for the Good-learner group (*p* > 0.005), state-value signals were nonetheless again found bilaterally in vmPFC (*xyz* = [-3.5, 30.5, -20.5], *t*_*14*_ = 3.65, *p* = 10^-3^, *k* = 18, SVC *p*_*FWE*_ = 0.137) among the Poor learners.
(TIF)

S6 Fig Good-learner group versus Poor-learner group. **(a)** The aforementioned lack of dorsal-striatal RPE signals among Poor learners was confirmed as part of direct contrasts of the Good-learner and Poor-learner groups with respect to the different parametric effects. First, the between-group contrast of SVPE signals revealed a cluster in the left dorsal striatum (*xyz* = [-15.5, 2, 14], *t*_*33*_ = 3.81, *p* < 10^-3^, *k* = 11) overlapping with that independently identified for the Good-learner group (*k* = 10) **(b)** Another region of the left dorsal striatum (*xyz* = [-17, 11, 8], *t*_*33*_ = 4.54, *p* < 10^-4^, *k* = 75) emerged from a direct contrast of the Good-learner and Poor-learner groups with respect to AVPE signals and again intersected with one of the clusters found for Good learner alone (*k* = 25). **(c)** Similarly, the lack of action-value signals in vmPFC among Poor learners was confirmed with a direct contrast that pointed to a cluster in bilateral vmPFC (*xyz* = [1, 33.5, -17.5], *t*_*33*_ = 3.57, *p* < 10^-3^, *k* = 20, SVC *p*_*FWE*_ = 0.126) overlapping with that independently identified as encoding action-value signals for the Good-learner group (*k* = 10).
(TIF)
