# Supplementary figures and images for: Distinct prediction errors in mesostriatal circuits of the human brain mediate learning about the values of both states and actions: evidence from high-resolution fMRI
Source: PLoS Comput Biol. 2017 Oct 19;13(10):e1005810. doi: 10.1371/journal.pcbi.1005810 (PMC5673235; doi:10.1371/journal.pcbi.1005810)

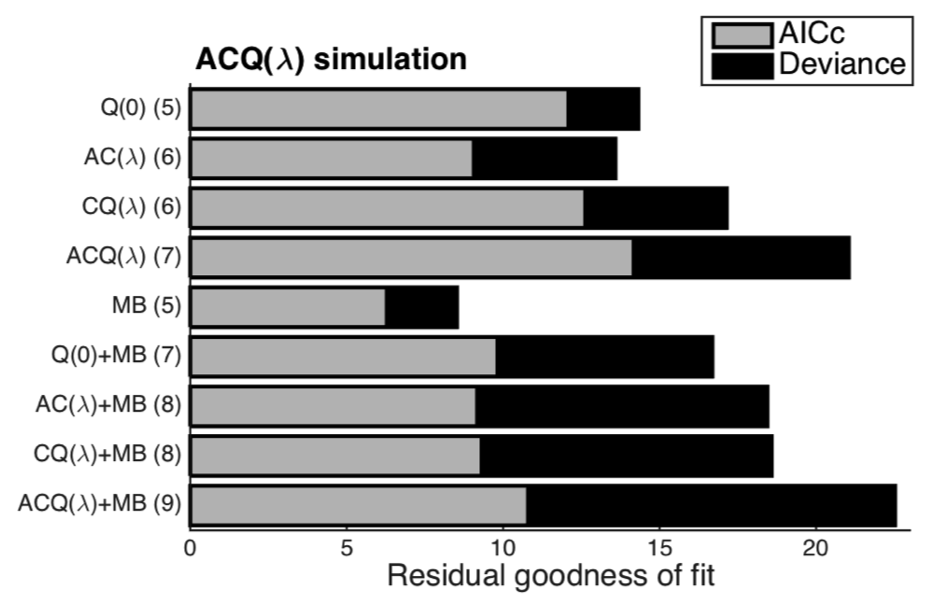

Supplement: S1 Fig — The model comparison reported in Fig 2A was replicated using artificial data that were simulated with the ACQ(λ) model as fitted for each subject but otherwise yoked to the empirical data set. Average goodness of fit relative to the outcome-insensitive hysteresis model across performing subjects is shown for each model tested with (light bars) and without (light and dark bars combined) a penalty for model complexity according to the AICc. A positive residual corresponds to a superior fit. As expected, only the ACQ(λ)+MB model—within which the actual model is nested—surpassed the actual model with respect to raw goodness of fit, but this overfitting was fully neutralized after correcting for model complexity. Degrees of freedom are listed in parentheses. (TIF) [file pcbi.1005810.s001.tif]

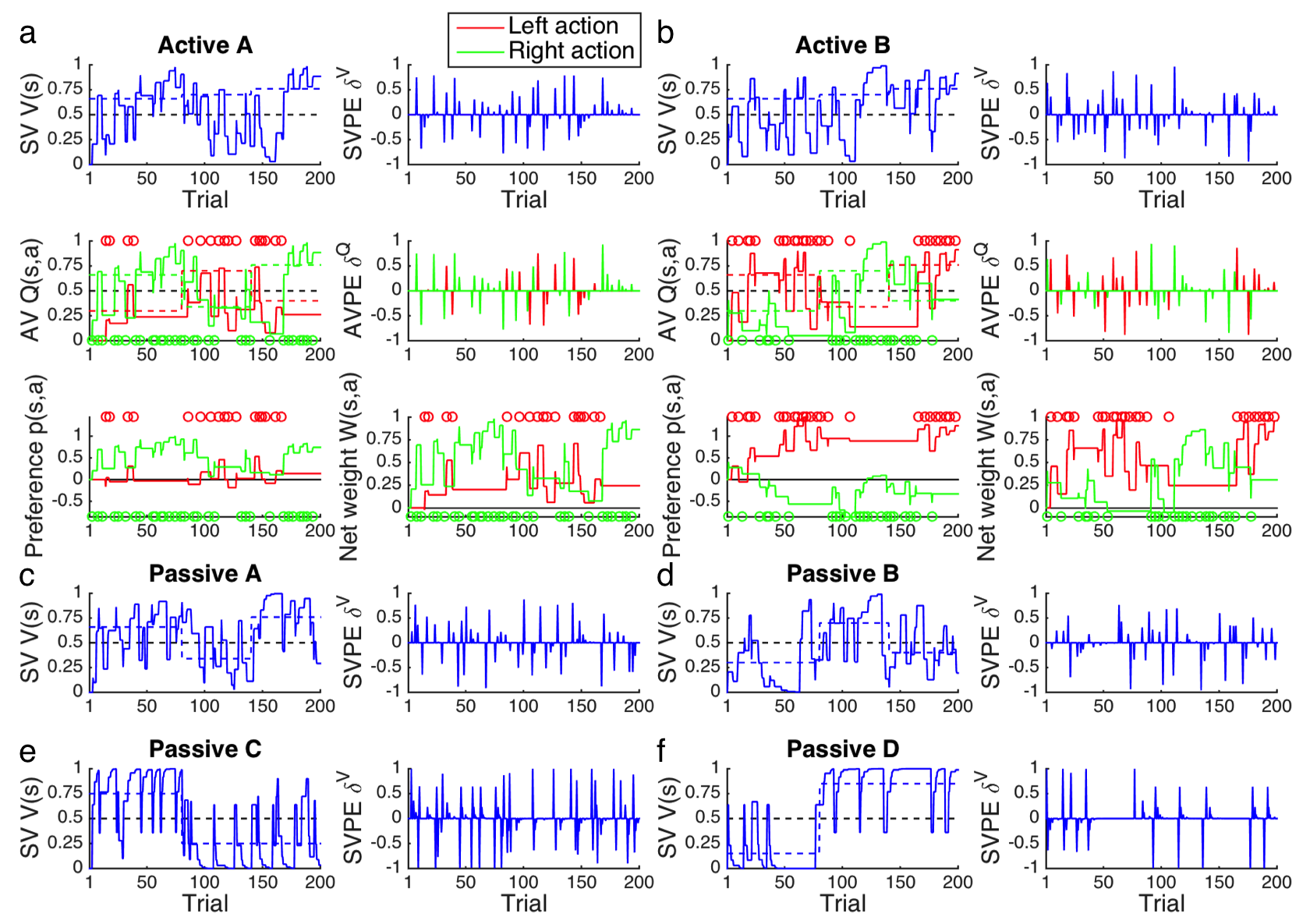

Supplement: S2 Fig — Representative dynamics of value signals and learning signals as generated by the ACQ(λ) model are Illustrated with the final subject from the Good-learner group. Fitted parameters were assigned as follows for this subject: α = 0.639, λ = 0.322, wQ = 0.857, τ = 0.197, β0 = -0.046, λβ = 0.976, and βR = 0.193. (a-b) The model’s estimates (solid lines) of state value (SV) Vt(s) as the probability of reward for the active states independent of actions are displayed in the upper-left corners of each panel along with empirical values (dashed lines) over the course of the experiment. Displayed in the upper-right corners are the state-value-prediction error (SVPE) δVt signals that for active states update not only the critic module’s state values Vt(s) but also the actor module’s relative action preferences pt(s,a), which are shown in the lower-left corners of each panel. As derived from the Q-learning component of the model, estimates of action value (AV) Qt(s,a) for the left and right options (red and green, respectively) are plotted at the left side of each panel along with empirical values. Each colored circle indicates an occurrence of the respective action. Adjacent to these plots on the right side of each panel are the time courses of the action-value-prediction error (AVPE) δQt signals updating the action values. Net action weights Wt(s,a) that integrate the aforementioned action preferences and action values are shown in the lower-right corners of each panel. (c-d) Time courses of state values and the SVPE are plotted for the first-stage passive states. (e-f) As plotted here, the SVPE for the second-stage passive states additionally updated representations for the first-stage states and actions via the eligibility trace. For this subject, a probability reversal at the second stage occurred before a probability reversal at the first stage. (TIF) [file pcbi.1005810.s002.tif]

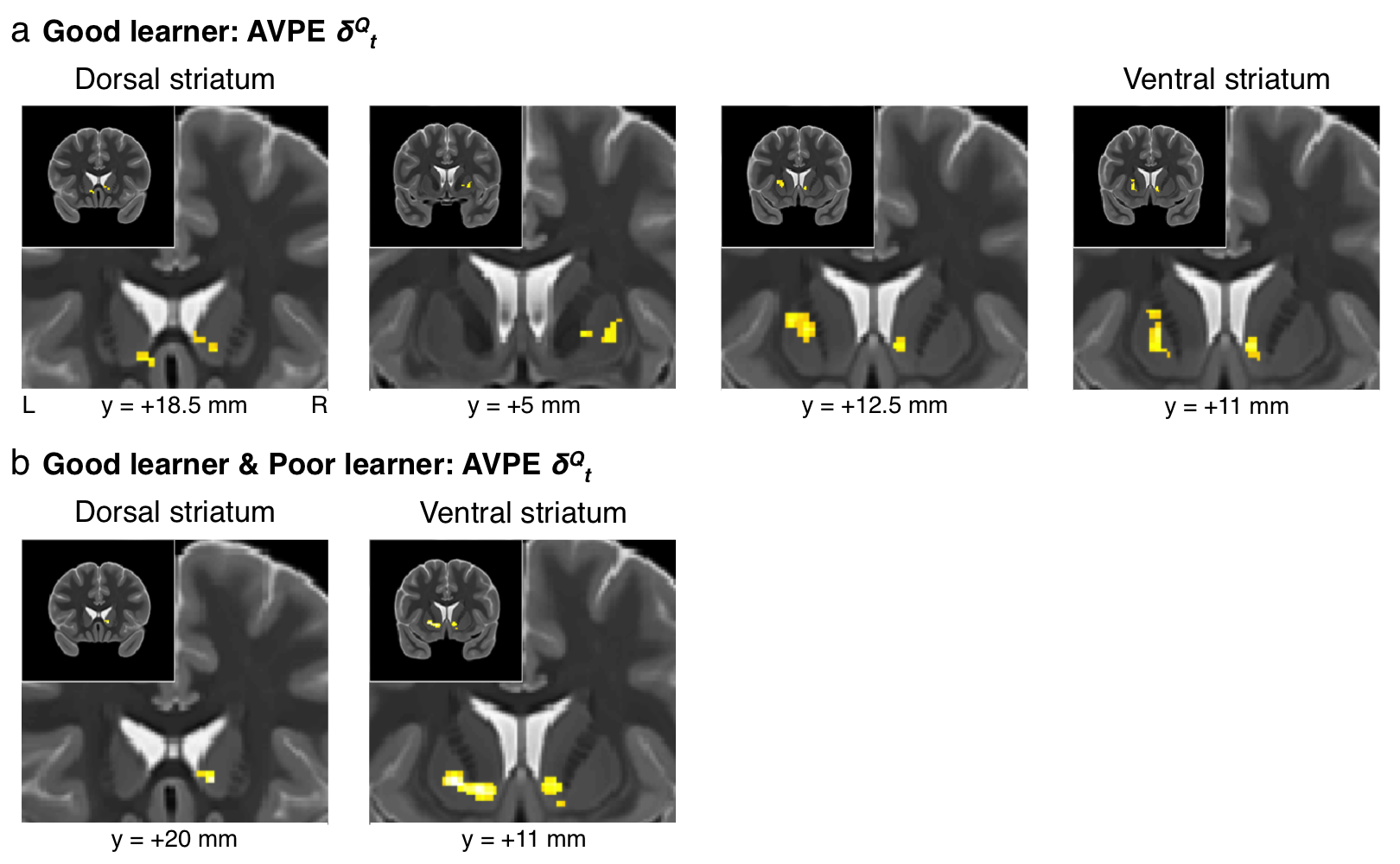

Supplement: S3 Fig — (a) For the Good-learner group, AVPE signals were identified throughout both the ventral striatum and the dorsal striatum. As with the aggregate analysis, the global peak of a cluster also within the ROI for the right ventral striatum (xyz = [8.5, 11, -2.5], t19 = 4.02, p < 10-3, k = 71, SVC pFWE = 0.064) was actually located in the dorsal striatum (xyz = [11.5, 20, -2.5], t19 = 4.13, p < 10-3). The corresponding anterior-caudate region in the left hemisphere (xyz = [-8, 18.5, -7], t19 = 3.53, p = 10-3, k = 14) was likewise engaged in this way. The anterior-caudate regions identified here are in close proximity to those reported for an instrumental RPE signal by O’Doherty and colleagues [19], both falling within 7.5 mm of the previously reported peak and its mirror-symmetric location. More caudally, AVPE signals were also observed in the right dorsal putamen (xyz = [28, 6.5, -1], t19 = 3.30, p = 0.002, k = 17). The last of these clusters distinguished the Good-learner and Poor-learner groups (S6B Fig) and was to be found in the left dorsal striatum (xyz = [-20, 11, 0.5], t19 = 4.12, p < 10-3, k = 58) for the most part but also extended somewhat into the ventral striatum. Otherwise, these results mostly aligned with those of the aggregate analysis of Good learners and Poor learners together. (b) Across all of these performing subjects, there were corrected significant results in the ventral striatum in both the left (xyz = [-12.5, 11, -5.5], t34 = 4.44, p < 10-4, k = 115, SVC pFWE < 0.05) and the right (xyz = [8.5, 12.5, -4], t34 = 3.87, p < 10-3, k = 108, SVC pFWE < 0.05) hemispheres as previously mentioned. Despite having local maxima within the ventral striatum, however, these same clusters also extended into regions of the dorsal striatum outside of the primary ROI with global peaks elsewhere in both the left (xyz = [-20, 11, -2.5], t34 = 4.55, p < 10-4) and the right (xyz = [11.5, 20, -2.5], t34 = 4.24, p < 10-4) hemispheres. (TIF) [file pcbi.1005810.s003.tif]

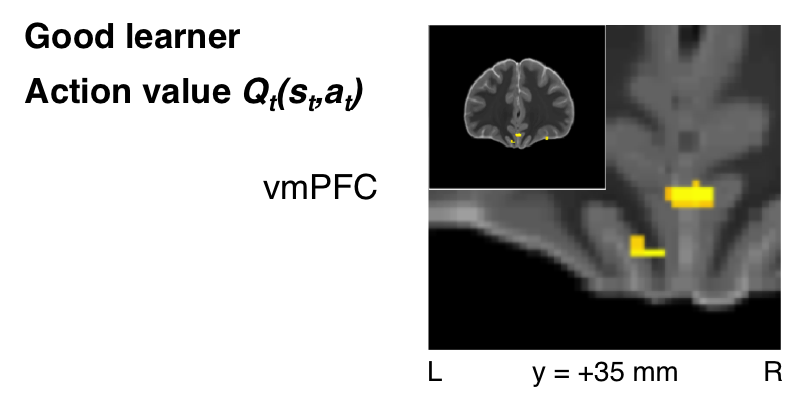

Supplement: S4 Fig — In addition to the separate types of RPE signals, separate types of value signals were evoked by the current paradigm. Among the Good-learner group, action-value signals were identified bilaterally in vmPFC (xyz = [1, 33.5, -17.5], t19 = 3.87, p < 10-3, k = 21, SVC pFWE = 0.086) as anticipated with marginal corrected significance. (TIF) [file pcbi.1005810.s004.tif]

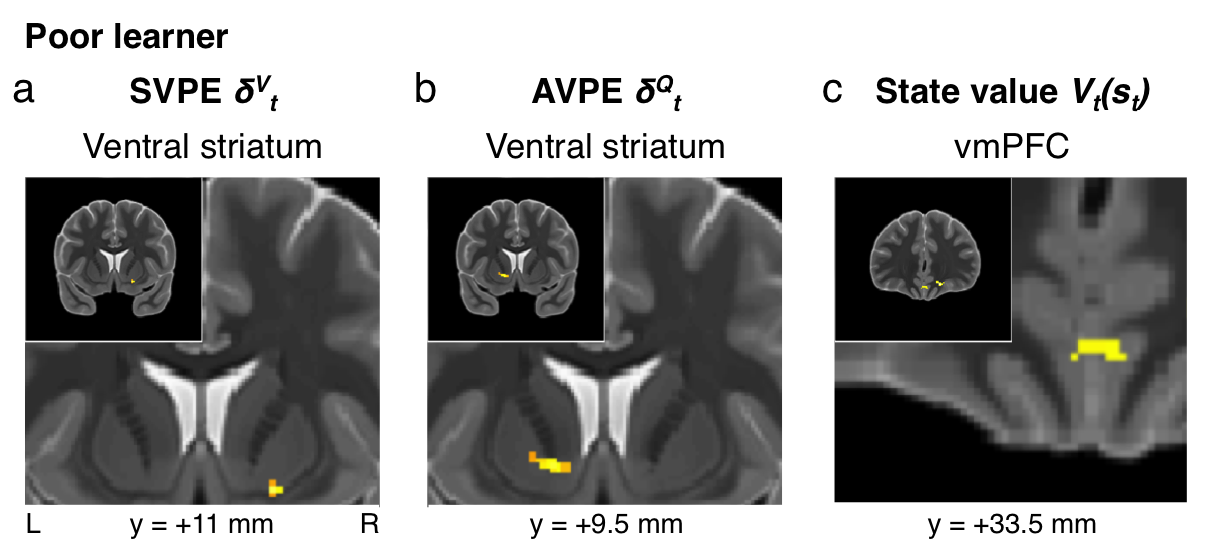

Supplement: S5 Fig — (a) For the Poor-learner group, the relevant neural signals were expected to be weaker as a reflection of the less robust learning evident in behavior. In line with this expectation, SVPE signals were only identified in the right ventral striatum (xyz = [19, 11, -11.5], t14 = 4.92, p = 10-4, k = 13). (b) Correspondingly, AVPE signals were limited to the left ventral striatum (xyz = [-12.5, 9.5, -5.5], t14 = 4.64, p < 10-3, k = 44, SVC pFWE = 0.056) among the Poor learners. (c) Although action-value signals were not observed in vmPFC at this statistical threshold for the Poor-learner group as for the Good-learner group (p > 0.005), state-value signals were nonetheless again found bilaterally in vmPFC (xyz = [-3.5, 30.5, -20.5], t14 = 3.65, p = 10-3, k = 18, SVC pFWE = 0.137) among the Poor learners. (TIF) [file pcbi.1005810.s005.tif]

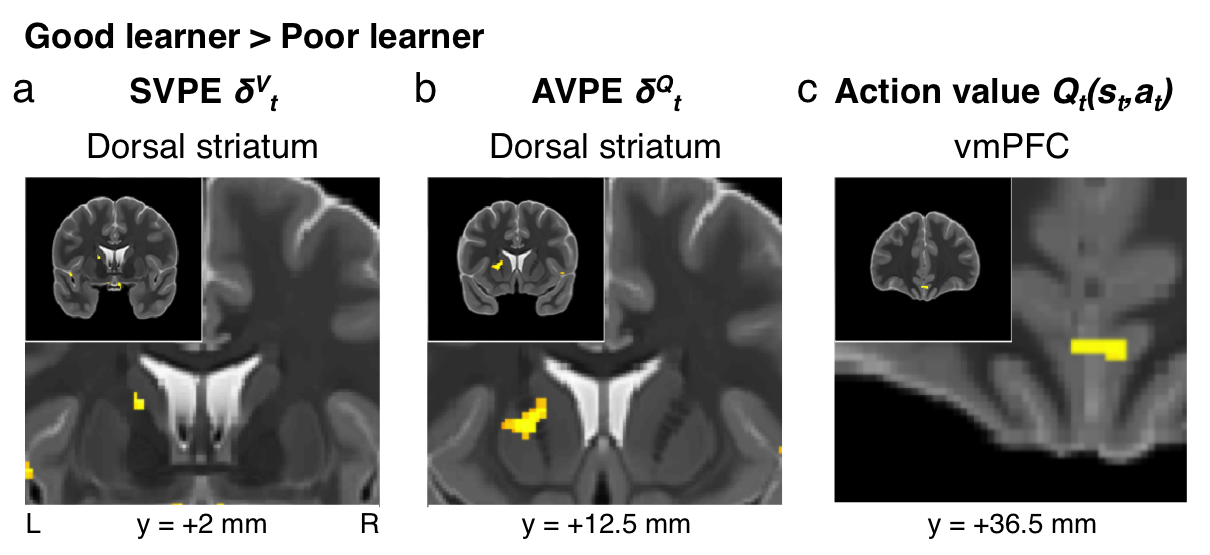

Supplement: S6 Fig — (a) The aforementioned lack of dorsal-striatal RPE signals among Poor learners was confirmed as part of direct contrasts of the Good-learner and Poor-learner groups with respect to the different parametric effects. First, the between-group contrast of SVPE signals revealed a cluster in the left dorsal striatum (xyz = [-15.5, 2, 14], t33 = 3.81, p < 10-3, k = 11) overlapping with that independently identified for the Good-learner group (k = 10) (b) Another region of the left dorsal striatum (xyz = [-17, 11, 8], t33 = 4.54, p < 10-4, k = 75) emerged from a direct contrast of the Good-learner and Poor-learner groups with respect to AVPE signals and again intersected with one of the clusters found for Good learner alone (k = 25). (c) Similarly, the lack of action-value signals in vmPFC among Poor learners was confirmed with a direct contrast that pointed to a cluster in bilateral vmPFC (xyz = [1, 33.5, -17.5], t33 = 3.57, p < 10-3, k = 20, SVC pFWE = 0.126) overlapping with that independently identified as encoding action-value signals for the Good-learner group (k = 10). (TIF) [file pcbi.1005810.s006.tif]
